# Development of a Bioinformatics Framework for Identification and Validation of Genomic Biomarkers and Key Immunopathology Processes and Controllers in Infectious and Non-infectious Severe Inflammatory Response Syndrome

**DOI:** 10.3389/fimmu.2020.00380

**Published:** 2020-03-31

**Authors:** Dong Ling Tong, Karen E. Kempsell, Tamas Szakmany, Graham Ball

**Affiliations:** ^1^Artificial Intelligence Laboratory, Faculty of Engineering and Computing, First City University College, Petaling Jaya, Malaysia; ^2^School of Science and Technology, Nottingham Trent University, Nottingham, United Kingdom; ^3^Public Health England, National Infection Service, Porton Down, Salisbury, United Kingdom; ^4^Department of Anaesthesia Intensive Care and Pain Medicine, Division of Population Medicine, Cardiff University, Cardiff, United Kingdom

**Keywords:** sepsis, SIRS, adult, pediatric, artificial neural network (ANN), gene interaction, biomarker

## Abstract

Sepsis is defined as dysregulated host response caused by systemic infection, leading to organ failure. It is a life-threatening condition, often requiring admission to an intensive care unit (ICU). The causative agents and processes involved are multifactorial but are characterized by an overarching inflammatory response, sharing elements in common with severe inflammatory response syndrome (SIRS) of non-infectious origin. Sepsis presents with a range of pathophysiological and genetic features which make clinical differentiation from SIRS very challenging. This may reflect a poor understanding of the key gene inter-activities and/or pathway associations underlying these disease processes. Improved understanding is critical for early differential recognition of sepsis and SIRS and to improve patient management and clinical outcomes. Judicious selection of gene biomarkers suitable for development of diagnostic tests/testing could make differentiation of sepsis and SIRS feasible. Here we describe a methodologic framework for the identification and validation of biomarkers in SIRS, sepsis and septic shock patients, using a 2-tier gene screening, artificial neural network (ANN) data mining technique, using previously published gene expression datasets. Eight key hub markers have been identified which may delineate distinct, core disease processes and which show potential for informing underlying immunological and pathological processes and thus patient stratification and treatment. These do not show sufficient fold change differences between the different disease states to be useful as primary diagnostic biomarkers, but are instrumental in identifying candidate pathways and other associated biomarkers for further exploration.

## Introduction

Sepsis is described as “*life-threatening organ dysfunction caused by a dysregulated host response to infection,”* leading to injury to healthy tissues, including those remote to the infection site (1, 2) and is part of a systemic inflammatory response syndrome group of conditions which can be infectious or non-infectious in origin. This can then progress to organ failure (such as acute kidney and acute lung injury) and septic shock if there is additional cardiovascular system involvement (3). Sepsis in its severe form typically results in admission to intensive care (ICU) and may be fatal (1, 4, 5). It is a major healthcare problem, killing over 44,000 people a year in the UK—more than lung, bowel, breast and prostate cancer combined (3, 6, 7). It costs the NHS £2.5 billion a year and is increasing in incidence (8). Patients present with clinical presentation and symptoms analogous to SIRS of non-infective origin (9), which is initiated by events such as trauma e.g., out of hours cardiac arrest (OOHCA). These conditions also exhibit a high degree of similarity in immune profile and they are hard to distinguish using conventional diagnostic methods (4, 9, 10).

There are a variety of causes of sepsis, including community and health care-related infections, and the condition commonly develops in patients with multiple risk factors, such as emergency surgery, diabetes and immunosuppression (6, 7). Regardless of the original initiating cause, sepsis develops to an inappropriate, dysregulated host inflammatory condition, in response to stimuli of infectious origin e.g., pathogen associated molecular patterns (PAMPs), such as endo- or exotoxins (11). These are recognized by pattern recognition receptors (e.g., Toll-like receptors or TLRs) and in sepsis ultimately lead to development of an inappropriate inflammatory response (12). These responses can be characterized using bioinformatic methods to determine signal-specific “fingerprints,” which can provide information on the underlying immune-pathological processes at work. These can be used to support diagnosis and inform patient management/therapeutic decisions (13).

The therapeutic options for sepsis have been extensively reviewed in the past and have been described as a “graveyard” for pharmaceutical companies (14, 15). Many treatments have been trialed but most of them failed to improve clinical outcomes in patients. Three notable inflammatory cytokines including tumor necrosis factor alpha (TNF-alpha), interleukin 1 (IL-1) and high mobility group box 1 (HMGB1) protein, have been assessed in clinical studies, but failed in clinical evaluation and are not now used as therapeutic interventions (14). The underlining challenge for development of improved immunomodulatory therapeutic options is hampered by a general lack of knowledge of the underpinning immune-pathological processes at work and/or identification of clinically useful biomarkers which can differentiate sepsis from SIRS. These would be useful, to aid correct diagnosis. Some progress has been made by other groups in the field in recent studies who have sought to better delineate the complex immunopathology of sepsis and develop discriminatory biomarker panels for disease stratification (16–22).

Here we describe a meta-analysis of previously published SIRS/sepsis and other infection datasets using artificial neural network (ANN) analyses, with additional interrogation of the input datasets using the bioinformatics package GeneSpring 12.5^TM^, to enable identification and assembly of SIRS/Sepsis immunopathology models and delineation of likely originator cell types. We have used similar methods to analyse gene expression data and delineate likely biomarker-associated cell types in a previously published Macaque model of Tuberculosis (23). The four main objectives of this study were to:

- identify a panel of gene expression profile biomarkers which distinguish sepsis patients from those who had clinical outcomes consistent with SIRS or the more severe septic shock
- investigate the immune-pathogenesis of these markers, primarily in sepsis
- compare these profiles to those observed in resolved SIRS
- uncover the likely cell types associated with key identified hub markers.

The combined data outputs from these objectives may provide valuable information for development of biomarkers for diagnostic purposes and provide valuable information on some of the key metabolic pathways and/or cell types involved in the underlying pathological processes.

## Materials and Methods

### Microarray Datasets

All microarray data used in this study were sourced from individual previously published datasets from the ArrayExpress database (24). These microarray data are available in the ArrayExpress website (http://www.ebi.ac.uk/arrayexpress/) under accession number E-GEOD-9960 [pathogen etiology not given (25, 26)], E-GEOD-28750 [pathogen etiology not given (27)], E-GEOD-6269 [a mixture of *Escherichia coli, Staphylococcus aureus*, and *Streptococcus pneumoniae* infections, in comparison with Influenza A (13)] and E-GEOD-13904 [pathogen etiology not given (28)]. Detailed information on the sample preparation on these datasets can be found in the original studies and on the ArrayExpress website. A total of 401 samples were obtained from these datasets and a summary of these datasets can be found in Table 1. These were all generated using the Affymetrix platform using two different gene chips: HG-U133A (E-GEOD-6269) and HG-U133_Plus_2 (E-GEOD-9960, E-GEOD-28750, and E-GEOD-13904).

**Table 1:** Summary of the datasets used in this study.

**GEOD dataset**	**References**	**Patient cohort**	**Sample distribution**							
			**Sepsis**	**SIRS**	**Post-surgical sepsis**	**Septic shock**	**Resolved SIRS**	**Healthy control**	**Gram-positive bacteraemia**	**Gram-negative bacteraemia**
E-GEOD-9960	(13, 25, 26)	Adult	45	25	–	–	–	–	–	–
E-GEOD-28750	(27)	Adult	10	–	11	–	–	20	–	–
E-GEOD-13904	(28)	Pediatric	52	27	–	106	24	18	–	–
E-GEOD-6269	(13)	Pediatric	–	–	–	–	–	–	37	26

### Data Pre-processing

Raw files of these microarray gene expression data (E-GEOD-9960, EGEOD-28750, E-GEOD-6269, and E-GEOD-13904) were downloaded from the ArrayExpress website and processed using the RMA (robust multi-array) method embedded in the Affymetrix Expression Console (29), to remove background noise, to normalize expression values of the probe sets and to provide an unbiased interpretation on the pathology of the selected markers. An auditing and filtration process was then applied, to remove unidentifiable probe entities and sets with multiple gene-associations. The expression values of the probe sets which associated to a single gene symbol were averaged and these were used as the final expression value for that gene. This filtering process yielded a total of 12,507 genes for microarray data stored in the HG-U133A chip (E-GEOD-6269) and 20,328 genes for HG-U133_Plus_2 (E-GEOD-9960, E-GEOD-28750 and E-GEOD13904). Samples were then categorized into 8 disease groups (i) sepsis (ii) SIRS (iii) septic shock (iv) resolved SIRS (v) healthy control (vi) gram-positive bacterial infection (vii) gram-negative bacterial infection (viii) post-surgical sepsis in two control patient cohorts—adult and pediatric (Table 1). Due to a low number of samples in the post-surgical sepsis group (11 samples), pathology analysis of this group is omitted in the rest of this study.

### Study Outline

**Figure 1:**
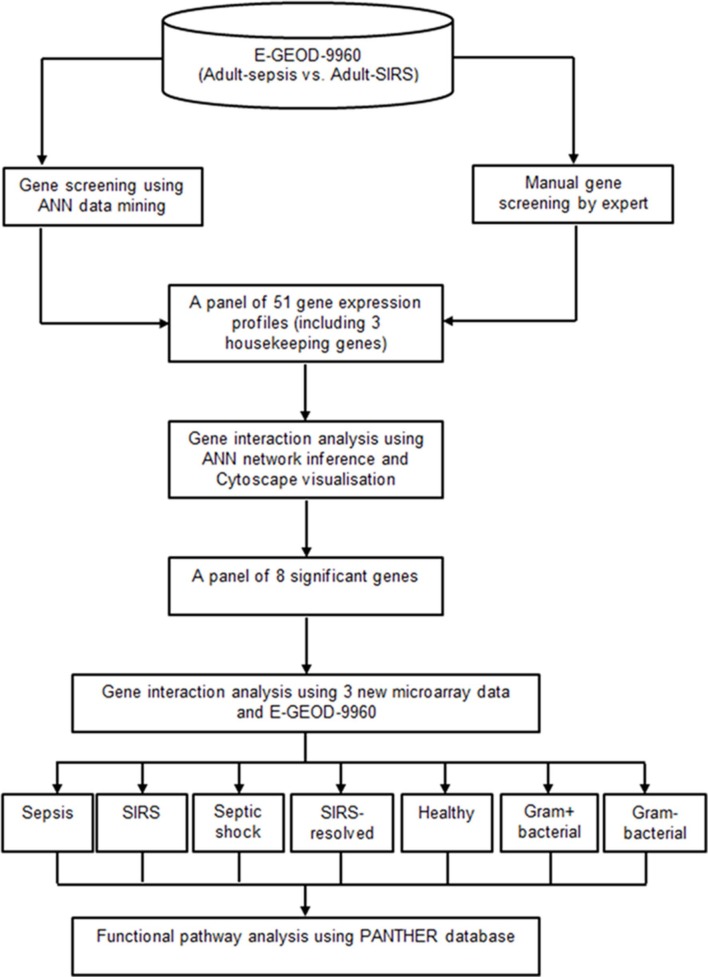
Analysis pipeline for this study.

A two-tier gene screening was conducted to identify a set of prominent markers segregating sepsis and SIRS in the adult cohort, using dataset E-GEOD-9960. Gene pathology analyses based on these markers was then performed, to reveal gene interaction activities and their pathways which associated specifically with sepsis, SIRS, septic shock, resolved SIRS or gram-positive and gram-negative bacteraemia. A panel of 51 genes (48 candidate genes and 3 housekeeping genes) associated to sepsis and SIRS were first selected based on the joint-decision obtained from ANN modeling (30) and an ontology-based review of other previously-published adult whole blood transcriptomic microarray data. A gene-gene interaction analysis based on these delineated gene subsets was performed using an inference model based on a three-layered backpropagation ANN algorithm (31). The gene interaction results were visualized using Cytoscape (31). A smaller set of eight key marker genes with strong interaction influences to other highly significant genes were identified using this process. Pathology analysis of these marker genes was performed with inclusion of three new microarray datasets (E-GEOD-6269, E-GEOD-28750 and E-GEOD-13904), comparing sepsis, SIRS, healthy control, resolved SIRS, septic shock, gram-positive and gram-negative using ANNs (31) and the PANTHER gene ontology (GO) database (32, 33). Figure 1 shows the analysis pipeline for this study.

### Preliminary Gene Screening

#### Test Set Screening for Candidate Genes Using ANN Data Mining Methods

Processed E-GEOD-9960 microarray data was downloaded from the ArrayExpress website. This contained 54,675 probe entities, which had been interrogated with RNA from a total of 70 patient blood samples, distributed into sepsis and SIRS specific groups (see Table 1). Using all probe entities, ANN-based predictive models were constructed to examine the prediction power of this dataset for differentiating sepsis patient samples from SIRS.

The ANN data mining algorithm used was developed by our group for mass spectra (34–36) and microarray analyses (30) and has been published previously (37). To identify disease-specific candidate markers, a 3-layered backpropagation ANN model with embedded exhaustive search strategy and cross-validation procedure was constructed. The sample set was randomly partitioned into 3 sets, i.e. training, test, and validation, in which the training set was used to train the model, the test set used for early stopping of the model and the validation set used to test the classification performance of the model. The ratio for the training, test and validation sets is 0.6:0.2:0.2, i.e., 60% of the samples are used for training purposes, 20% for testing and the remaining 20% for validation. To avoid any bias on reported results, samples were randomly re-shuffled 50 times for training, test, and validation purposes.

For the ANN architecture, a structure of 1-2-1 was applied. An input node representing a unique probe set in the dataset, two hidden nodes in the hidden layer and an output node predicting sample class. As an exhaustive search strategy was applied, a new probe set identification was selected as the input node in the input layer each time a new network model was created. A sigmoid activation function was utilized in the algorithm. Three hundred epochs were used for the training process and 100 epochs for the testing window, stopping if the mean square error (MSE) failed to improve <0.01 over the window. A single input node was deliberately applied to ensure that all probe sets in the data were thoroughly examined by the ANN. Finally, the probe sets were ranked based on their standard residual errors (SRE), computed using a multiple regression method, which are equivalent to fold-change differences in other gene expression profile analysis methods.

To identify genes for which probe sets were highly ranked in truncated *p*-values, a stringent filtering process was applied to remove probe sets that did not meet any one of the following criteria: (i) a single probe set should have only associated to a single gene symbol; (ii) all probe sets to which a similar gene associated to should have a consistent regulation response pattern in a similar disease group; and (iii) any unlabelled probe set (i.e., unknown gene symbol) should contain sufficient information for finding the associated gene symbol in a BLAST search. It was hypothesized that a “true” marker should be reproducible and exhibit a consistent differential regulation response pattern in individuals from a similar disease group. A panel of 48 candidate markers and three housekeeping genes were identified and are summarized in Supplementary Information S2, Table S1.

#### Gene Interaction Analysis Using ANN Inference (ANNI) Algorithm

Using the selected candidate biomarker genes from primary gene screening, a network inference model based on a 3-layered backpropagation ANN algorithm called ANNI (38, 39) was used to model the molecular interactions between genes identified for sepsis and SIRS. ANNI is a bespoke algorithm developed by our group to model the relationship between genes, to gain an in-depth understanding on how these molecules interact and to identify new relationships between these molecules by iteratively calculating the influence that multiple genes may have upon a single entity. This algorithm hypothesized that expression (i.e., up- or down-regulation) of a biomarker can explain, using the remaining biomarkers in the gene pool, if these biomarkers are able to explain one particular categorical outcome (i.e., a disease status). This explores the influences of all biomarkers among themselves and provides a complete view of all the possibilities for network interactions between all biomarkers.

In brief, a new ANN interaction model was constructed each time a gene in the gene pool was selected as an output node and was omitted from the pool. The remaining genes (*N* − 1) were then used to predict the omitted gene and the network weights of the trained model were stored. The process continues until all the genes in the pool were used as output. The weights of all the trained models were then averaged and served as the final interaction results to describe the interaction between genes, such as the intensity of the relationship between a source gene and a target gene and the nature of their relationship (e.g., stimulatory or inhibitory). Further information on this algorithm can be found in our previous study (40).

#### Interactome Network Map Visualization Using Cytoscape

Gene interaction results were displayed visually using the Cytoscape software platform (41). Using the gene interaction output of the candidate gene selection analyses, genes with strong interaction influence on other genes were chosen for further analysis. Gene pathology analysis based on these gene entities was interrogated to segregate sepsis from SIRS, septic shock, resolved SIRS, gram-positive and gram-negative bacteraemia and healthy controls.

### Functional Pathway Analysis Using the PANTHER Database

Pathway analysis for the selected marker genes was performed by mapping these genes onto the PANTHER (Protein Analysis Through Evolutionary Relationships) database (Version 8).

### Parametric ANOVA and Similar Entity Analyses Using Agilent GeneSpring 12.5

Data output files from E-GEOD-9960, E-GEOD28750, E-GEOD-6269, and E-GEOD-13904 were imported into GeneSpring GX™ v12.5 (42). Data were normalized to the 75th percentile followed by median baseline transformation, according to default settings. Statistically significant features from a predefined entity list (Supplementary Information S3, Table S3.1). were identified using one-way ANOVA analysis across all entities and time-points, using the Benjamini-Hochberg False Discovery Rate (BH-FDR) (43), with multiple testing corrections at a cut-off *p* ≤ 0.05. Associative relationships between key candidate and hub genes and other gene entities were conducted using the similar entities function using a cut-off of >0.7. All further analyses were conducted using hierarchical cluster analysis, heat map, and other functions in GeneSpring GX v12.5, using default settings.

## Results

### Gene Screening Analyses

The rank distribution of all 54,675 probe sets based on regression errors is shown in Supplementary Information S1, Figure S1. A total of 11,846 probe sets were found with SRE > 1.0 (5,445 probe sets) and SRE < -1.0 (6,401 probe sets). Amongst these probe sets, 1,647 with SRE > 2.0 and 139 with SRE < -2.0. The filtering criteria yielded a total of 14,872 unique genes. Amongst these genes, 7,589 genes (inclusive of 1,501 genes with SRE > 1.0) exhibited an up-regulation pattern in sepsis and the remaining 7,283 (1,823 with SRE < −1.0) exhibited up-regulation in SIRS. The full gene datasets were then integrated with empirically-derived functional ontology gene lists from previously published literature [from (44–49)], which yielded a final selection list of 48 candidate genes. A summary of these is provided in Supplementary Information S2, Table S1, showing the product of *p*-values using the rank order from ANN data modeling and Student *t*-tests, computed using the BH-FDR. Of these, 22 genes were found to be relatively upregulated in Sepsis vs. controls and the remaining 26 upregulated in SIRS vs. controls. High median expression values were observed in both the adult Sepsis and adult SIRS groups (Supplementary Information S1, Figures S2, S4), demonstrating that overall these genes had higher expression in these two groups compared with any other group in the study.

### Selection of Housekeeping Genes

Selection of housekeeping genes is a crucial process for customizing microarray gene expression data (50), as they are used to normalize mRNA levels between different samples in qPCR. A housekeeping gene should be consistently expressed in all gene expression samples, irrespective of which disease group the sample belongs to and have poor prediction power in stratifying samples. Inappropriate selection of housekeeping genes could bias gene expression differences between different groups and samples during analysis, rendering array data incomparable. Selection of reference genes may be study or disease specific, as there is variation in identification of useful candidates even in a single disease area (51). A total of 129 candidate housekeeping genes, including several commonly used entities e.g., GAPDH (52–56), were investigated in this study. The stability of these genes was ranked according to predictive power of the genes in stratifying adult sepsis from adult SIRS and their *p*-values. HMBS (predictive *p* = 0.82, *t*-test = 0.73), TBP (predictive *p* = 0.60, *t*-test = 0.32) and ALAS1 (predictive *p* = 0.44, *t*-test = 0.20) were found to be the most invariant and thus selected as suitable housekeeping genes for further expression study data analysis (given in Supplementary Information S2, Table S2). These commonly used reference genes may be more specific for SIRS/Sepsis datasets and useful for normalization purposes in future development of diagnostic assays.

### Identification of Key Marker Genes

**Figure 2:**
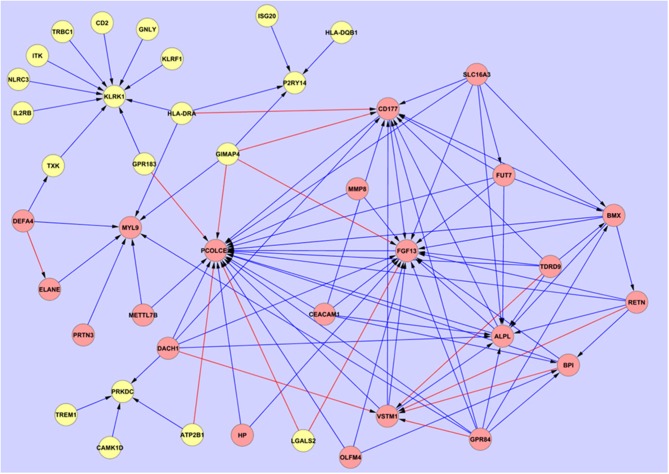
Overarching network interaction map between the hub biomarkers and other significant disease-associated entities. Source 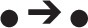 Target: 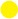 Entities which show relative upregulation in SIRS compared with sepsis: 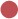 Entities which show relative upregulation in sepsis compared with SIRS: 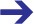 Inhibitory interaction: 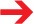 Stimulatory interaction.

Using the 48 gene expression markers identified in the initial gene screening process, interactions between these markers were modeled (Figure 2). Eight prominent markers which showed higher interaction activities with other genes were identified, which are KLRK1, MYL9, PCOLCE2, CD177, FGF13, SLC16A3, GPR84, and TDRD9. These genes may reflect 8 core biological activities at work in the respective disease processes and may also provide important information for connections to other genes (Table 2). These may have other alternative functions dependant on cell type, relative disease condition or homeostasis, in addition to regular ascribed cellular functions.

**Table 2:** Primary and alternate cellular functions of hub gene entities.

**Hub gene ID**	**Name**	**Gene annotation**	**(1) Primary Function (2) Alternate Function**	**References**
CD177	CD177 molecule	A cell surface glycoprotein exclusively expressed by neutrophils, it plays a role in adhesion and exvagination of neutrophils from the peripheral blood	(1) Delineates neutrophil subsets and regulates transmigration across the endothelium through the interaction with platelet endothelial cell adhesion molecule-1	(57–63)
FGF13	Fibroblast growth factor 13	A potent factor involved in a variety of biological processes including cell growth and death, embryonic development and other cellular processes	(1) Regulates cell proliferation, differentiation and morphogenesis and invasion by binding to the extracellular domain of cell surface receptors (2) Microtubule-stabilizing protein regulates neuronal polarization and migration	(64–72)
GPR84	G protein-coupled receptor 84	A putative pro-inflammatory receptor that plays a critical role in a variety of physiological homeostasis activities. This receptor is activated by medium-chain fatty acids and may be associated with chronic inflammation	(1) Functions as an enhancer of inflammatory signaling in macrophages, up-regulated in endotoxin-tolerant macrophages - modulates responses to TNF-α (2) Recruitment in neutrophils	(73–81)
KLRK1 (NKG2D)	Killer cell lectin-like receptor subfamily K, member 1	Activating receptor expressed by immunogenic cells including all NK cells and subsets of T cells. It plays an important role in immune system by serving as a major recognition receptor for detecting and eliminating transformed or infected cells	(1) Activating receptor on natural killer (NK) and T-cells and binds a diverse panel of polymorphic ligands encoded by the MIC and RAET1 gene families (2) Promotes B1a cell development and protection against bacterial infection	(82–84)
MYL9	Myosin, light chain 9, regulatory	A structural component of muscle that plays a vital role in growth and development of smooth muscle, associated to the contractile activities in smooth muscle and non-muscle cells. Other regulatory functions	(1) Structural component of muscle fiber (2) Regulation of platelet and macrophage development (3) alternate pathway for CD3 expression on T-cells	(85–92)
PCOLCE2	Procollagen C-endopeptidase enhancer 2	A glycoprotein that plays a central role in physiological activities including cell signaling processes (i.e., cell adhesion, cell communication and transport), cellular, developmental and metabolic process and also in development or function of the immune system in response to internal/invasive threats	(1) Mycocardium collagen deposition, bone morphogenesis (2) High density lipoprotein synthesis (3) Immune cell extracellular matrix formation	(93–96)
SLC16A3	Solute carrier family 16 (mono-carboxylate transporter) member 3	A transmembrane protein that transports lactate and mono-carboxylates (both endogeneous and exogenous) across the cell membrane	(1) Monocarboxylate transporter 4 that shuttles lactate out of the cell, may be upregulated during shift to accelerated glycolysis during critical illness (2) Other functions may include a role in maintaining Colonic Homeostasis	(97–102)
TDRD9	(Tudor domain containing 9)	A putative protein that plays a central role during spermatogenesis and other metabolic processes	(1) Transposon silencing in the male gonad (2) Suppression of DNA repair genes	(103–109)

### Regulation Response Pattern Analysis

Using the healthy controls group as baseline control to determine the regulation response of these markers, fold change expression differences were determined (given in Supplementary Informations S1 and S2; Figure S3 and Table S2). Significant up-regulation patterns were observed in both sepsis and SIRS. From these data it was observed that the expression levels of these 8 markers are higher in all disease groups (Supplementary Information S1, Figure S4), compared with healthy controls, except for KLRK1 which shows little change in adult SIRS and sepsis and is downregulated in pediatric SIRS, sepsis and septic shock. Significantly higher fold change differences were observed in disease groups compared with controls in the adult compared to the pediatric group, especially for FGF13 and PCOLCE2. PCOLCE2 is also downregulated in pediatric gram-negative infection.

### Overlapped Genes and Pathway Analyses

Further investigation on the performance of these hub markers in delineating individuals with sepsis, SIRS, septic shock, resolved SIRS, gram-positive or gram-negative bacteraemia or healthy controls was performed. This revealed unique and overlapped gene interactions and pathway-associations for these groups. The eight core hub genes show strong interaction influences to other linked associated genes (Figure 2). CD177, FGF13, KLRK1, MYL9 and PCOLCE2 act as target hub genes to interconnect to their neighboring genes and GPR84, SLC16A3 and TDRD9 play important roles as secondary source genes, targeting their specific, associated genes. To more fully understand these hub gene interactions, these eight markers underwent gene classification analyses in each of the six different disease groups, to identify a set of highly predictive genes that could influence the interaction pathways between these markers. As markers GPR84, KLRK1, and TDRD9 were not represented in dataset E-GEOD-6269 (Affymetrix chip HG-U133A), interaction profiling of these genes was omitted from the gram-positive and gram-negative bacterial infection group dataset. Using the exhaustive ANN algorithm described above, a total of 740 new genes (10 new genes × 8 markers × 8 groups + 10 new genes × 5 markers × 2 groups) were revealed. Low predictive errors (mean error = 0.1578) were observed for each of these hub markers across all the groups (Supplementary Information S2, Table S3).

Amongst these newly selected genes, some overlapped between groups (Supplementary Information S2, Table S4). These entities may also participate in activating metabolic pathways (pathway associations for the disease groups are given in Supplementary Information S2, Table S5). High numbers of overlapped genes were found in pediatric septic shock compared with pediatric sepsis, suggesting that these two clinical manifestations of sepsis have similar response pathways and biological processes, as expected. These overlapped genes are MYL9 (whose primary function is in muscle development or cell movement but has a secondary function in regulating platelet development) and immune/inflammatory response pathways (PCOLCE2, GPR84 and CD177). A small proportion of the overlapped genes participated in different activities in other cellular processes (FGF13, TDRD9) suggesting a possible way to stratify these groups based on these activities. In addition to the overlapped pathways, there is also evidence presented that some pathways are uniquely associated to a specific disease group. However, stability analyses for these pathways in the respective disease group using larger patient cohorts collected at different time points are needed. This is to ensure such pathways are in fact unique for each of the disease group and that they can be used as candidate pathways to identify different progressed stages in both SIRS and sepsis, which may better inform patient management and care. The second observation suggests a possible impairment in cell growth and death and other cellular processes in adult SIRS.

### Regulation Response and Gene Interaction Analyses

These hub genes were further investigated for their ability to stratify sepsis from SIRS, septic shock, resolved SIRS, gram-positive, or negative bacteraemia. The majority of these genes showed inhibitory responses when interacting with the other genes. Genes expressed in SIRS demonstrated distinct interaction responses when interacting with those also highly expressed in SIRS. All the SIRS-expressed genes showed stimulatory responses when interacting with genes that were highly expressed in sepsis. This stimulatory response appeared inhibited when they interacted with one another. Stimulatory interaction responses were observed in most of the gene clusters in septis, SIRS, septic shock, resolved SIRS, and bacteraemia. The latter gram-positive/gram-negative bacteraemia disease group exhibited few interactions between the hubs and complex, unique interaction signatures. The results summary is given in Supplementary Information S4 and interaction maps in Supplementary Information S5, this group was not analyzed further.

**Figure 3:**
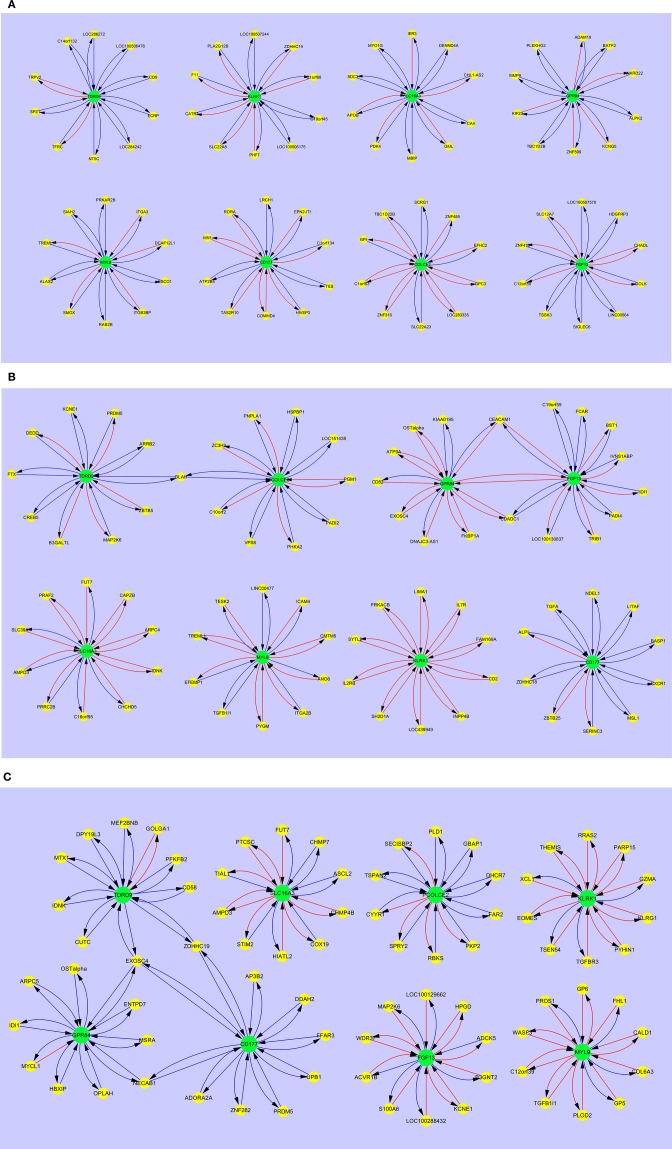
Interaction maps between hub biomarkers, entities and hub nodes; **(A)** healthy control adult **(B)** SIRS adult **(C)** sepsis adult. Source 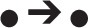 Target: 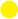 Predictive gene: 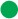 Candidate gene: 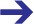 Inhibitory interaction: 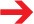 Stimulatory interaction.

**Figure 4:**
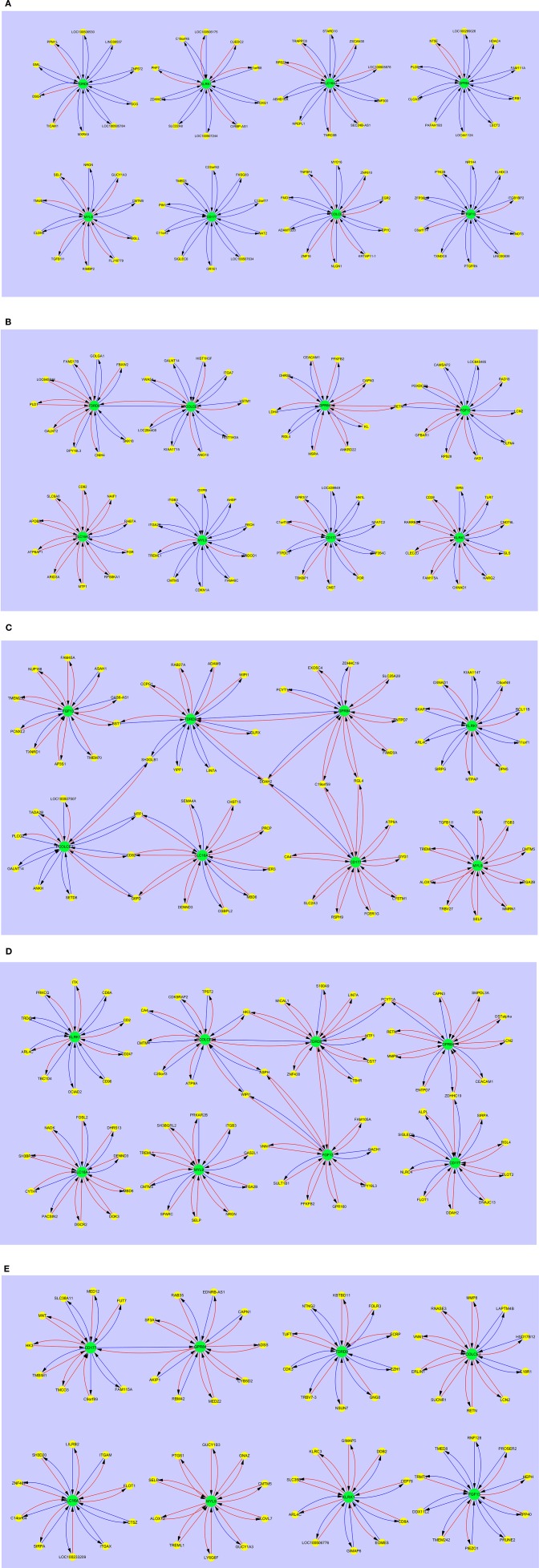
Interaction maps between hub biomarkers, entities and hub nodes; **(A)** healthy control pediatric **(B)** SIRS pediatric **(C)** SIRS-resolved pediatric **(D)** sepsis pediatric **(E)** septic shock pediatric. Source 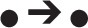 Target: 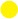 Predictive gene: 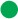 Candidate gene: 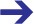 Inhibitory interaction: 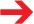 Stimulatory interaction.

Interaction maps were generated from gene interaction analyses using Cytoscape and are given in Figures 1–4. These show the specific regulation response pattern and bi-directional interaction gene interactomes for the selected 8 markers in all 10 disease groups. Amongst the clusters in the maps, cluster MYL9, GPR84, and SLC16A3 showed uni- and/or bi-stimulatory signals in most groups, with the exception of adult sepsis which exhibited inhibitory signals. The majority of these clusters were not connected in most disease and control states, except for adult sepsis and adult SIRS with the gene clusters for TDRD9, CD177, GPR84, PCOLCE2, FGF13 and SLC16A3 interconnected at various points, either directly or through overlapped gene connections (Supplementary Information S2, Table S6).

#### Gene Interaction Patterns in Adult SIRS and Sepsis and Healthy Controls

Figure 3 shows the interaction maps of the core hub entities in (a) adult healthy controls (b) adult SIRS and (c) adult sepsis. In the healthy control group (Figure 3A), it can be seen that there is interaction of the hub entities with a number of other gene entities, most of which is inhibitory in character, as indicated by the blue arrows. There is a small amount of stimulatory activity, particularly for PCOLCE2 and CD177. There is little interaction between the 8 hub-centered activities, which may indicate a state of relative homeostasis. In the adult SIRS group (Figure 3B), it can be seen that the complement of genes associated with each hub gene varies from those observed in healthy controls and there is significantly more stimulatory activity between the hub genes and the interacting gene entities, particularly for KLRK1. There is also increased interaction between the hubs, indirectly between TDRD9 and PCOLCE2 through OLAH, directly between GPR84 and FGF13 and indirectly through CEACAM1 and CDADC1. The interaction maps for the sepsis group (Figure 3C) show a similar pattern to those for SIRS, i.e., specific changes in the complement of genes associated with each hub gene and significantly increased stimulatory interactions for KLRK1, FGF13, and MYL9. There are interactions between the GPR84, CD177, and TDRD9-centered hubs, through EXOSC4, ZDHHC19, and NECAB1 which appear profoundly inhibitory in nature, in contrast to the healthy controls. The MYL9-centered hub shares entities TREML1 with adult healthy controls and TGFB1l1 with adult sepsis.

Comparing the gene interaction profiles between adult sepsis and adult SIRS in Figures 3B,C, different gene interaction activities were observed. In adult sepsis, genes TDRD9, GPR84, and CD177 interacted via overlapped genes EXOSC4 (TDRD9 ↔ GPR84 ↔ CD177), ZDHHC19 (TDRD9 ↔ CD177) and NECAB1 (GPR84 ↔ CD177). TDRD9 and GPR84 in adult SIRS, interacted however with FGF13 (GPR84 ↔ FGF13) and PCOLCE2 via the overlapped gene OLAH (TDRD9 ↔ PCOLCE2). The interactions in sepsis patients suggest possible co-activation in inflammation actions.

#### Gene Interaction Patterns in Pediatric SIRS, Sepsis, Severe Sepsis, Resolved SIRS, and Healthy Controls

Figure 4 shows the interaction maps of the core hub entities in (a) pediatric healthy controls; (b) pediatric SIRS; (c) pediatric sepsis; (d) pediatric severe sepsis (septic shock) and (e) pediatric resolved SIRS. A similar pattern of mainly inhibitory interactions of core hub entities with other gene entities was observed in pediatric healthy controls (Figure 4A) and these differ in the main from those observed in adult healthy controls, except those centered around KLRK1 i.e., C1ORF68, C19ORF45, LOC100506175, LOC100507244, PHF7, SLC22A8, and ZDHHC19, which are shared. SIGLEC6 appears to be variably connected with FGF13 in the adult and CD177 in the pediatric controls.

In pediatric SIRS (Figure 4B), as seen with the adults, the complement of genes associated with each hub gene alters, there is significantly more stimulatory activity between the hub genes and the interacting gene entities, particularly for KLRK1 and SLC16A3. There is also increased interaction between the hubs TDRD9 and PCOLCE2, however directly in this case and between GPR84 and FGF13 indirectly through RETN. CEACAM1 interacts with GPR84, but not FGF13 in pediatric SIRS. The only hub which exhibits shared entities other than CEACAM1 is the MYL9 hub, which shares CMTM5, ITGA2B and TREML1 with the adult dataset.

The interaction maps of the core hub entities in resolved pediatric SIRS are shown in Figure 4C. In addition to alterations in the complement of most hub-centered entities, with the exception of the MYL9-centered hub, the connections between the TDRD9 and PCOLCE2 and GPR84 and FGF13-centered hubs appear now dissolved and there is a single connection between the CD177 and GPR84-centered hubs. There are few shared entities between the core entity-associated hubs, with the exception of MYL9 which shares CMTM5 and SELP with pediatric controls and SIRS and TREML1 with pediatric SIRS.

In pediatric sepsis (Figure 4D) there are again alterations in the complement of genes associated with each hub gene and significantly increased stimulatory interactions for nearly all hubs, with the exception of KLRK1 and PCOLCE2. TDRD9 and SLC16A3 show more modest stimulatory activity. There are interactions between the TDRD9-centered hub and GPR84, directly and through DDAH2, with CD177 through DDAH2, with FGF13 through BST1 and with PCOLCE2, through SH3GLB1. There is interaction of GPR84 with CD177 through C19orf59 and RGL4 and with SLC16A3 and PCOLCE2, through MTF1, CD82, and G6PD. There are few shared hub-associated entities between adult and pediatric Sepsis, with the exception of GPR84-associated EXOSC4 and ENTPD7, CD177-associated DDAH2 and MYL9-associated TGFB1l1.

Figure 4E shows the interaction maps of the core hub entities in pediatric severe sepsis (septic shock). The pattern is similar to that for pediatric sepsis with connections between nearly all hubs with the exception of KLRK1, SLC16A3, and MYL9. There are also changes in the complement of genes associated with each hub gene and increased stimulatory interactions, compared with the healthy control profiles. There are interactions between the TDRD9-centered hub and PCOLCE2, directly and through HK3, with the FGF13-centered hub directly and the GRP84-centered hub through PCYT1A. There is connection between the GRP84-centered hub to the CD177-centered hub through ZDHHC19.

Gene entities shared between sepsis and severe sepsis response hubs are; TDRD9 – LIN7A, GPR84 – ENTPD7, PYCT1A and ZDHHC19, CD177 – DDAH2 and RGL4, SLC16A3 – DENND3 and MBD6, MYL9 – CMTM5, ITGB3, ITGA2B, NRGN, SELP and TREML1. The only entities shared with pediatric healthy controls are CMTM5, NRGN and SELP and are also associated with the MYL9-centered hub.

### PANTHER Functional Pathway Analysis

A total of 82 PANTHER pathways were found containing 333 hits, based on the 748 gene identified previously (see section Overlapped Genes and Pathway Analyses and Supplementary Information S1, Figure S5). The healthy groups in both the pediatric and adult cohorts had a much lower number of pathway hits than all disease groups in general, as perhaps expected. These pathways were grouped into 11 major themes. The majority of the gene hits were derived from the pediatric group (pediatric septic shock(p), 57 hits, 17.1%), SIRS-resolved (SIRS(p), 49 hits, 14.7%), pediatric gram-positive (gram +bacterial(p), 43 hits, 12.9% and also adult SIRS (SIRS(a), 35 hits, 10.5%). These were related to one or more of the following themes: signal transduction, G protein–related, growth and development and also immune system. High numbers of signal transduction hits were also found in the adult group (13 hits in sepsis and 17 in SIRS) when compared to the same disease group in the pediatric cohort (6 in sepsis and 6 in SIRS). In the pediatric cohort, a high number of signal transduction hits were also observed in SIRS-resolved and septic shock. In addition, the G protein-coupled receptor pathway was found to have a large number of identified hits in the pediatric cohort, including SIRS-resolved (11 hits), septic shock (10 hits), and gram-positive bacteraemia (9 hits). In general, a higher number of hits were found in both patient sepsis groups when compared to the SIRS groups in the growth and development pathway. The immune system pathway, in contrast, had a higher number of hits in the SIRS and gram-positive bacteraemia groups than in the sepsis group. All disease groups except the adult SIRS group, were found to have gene hits associated with neuro-degenerative disorders.

### Similar Entities Analysis

Many of the eight key hub entities have ascribable functions and can be assigned to a particular cell type with some confidence, e.g., neutrophil-specific CD177. However, it was not apparent which cell type(s) were associated with expression of some of the other hub entities. To ascertain which of the entities could be associated with other cell delineating markers further parametric and similar entity analyses were conducted. Data were imported into GeneSpring 12.5 from the respective repositories as outlined above, normalized to the 75th Percentile and baseline transformed to the global median. ANOVA or *T*-test parametric analyses were conducted on each dataset and the results then ranked on *p*-value, from highest to lowest (given in Supplementary Information S6–S9; Tables S6.1, S7.1, S8.1, S9.1).

Using a focused biomarker gene list, which includes cluster of differentiation (CD) and other cell-associated biomarkers (Supplementary Information S3), similar entity analyses were conducted for each of the 8 hub markers [using default settings (Euclidean) and a distance-based similarity (or covariance) cut-off value ≥ 0.7], to establish potential cell-type associations. Using this analysis, a number of gene entities were found to co-associate with hub genes CD177, FGF13, GPR84, KLRK1, MYL9, PCOLCE2, and TDRD9 in datasets E-GEOD-6269 (Supplementary Information S6, Tables S6.2–S6.9), E-GEOD-28750 (Supplementary Information S7, Tables S7.2–S7.9), E-GEOD-13904 (Supplementary Information S8, Tables S8.2–S8.9) and E-GEOD-9960 (Supplementary Information S9, Tables S9.2–S9.9). The entities associated with the hub genes which are shared between the 4 datasets are summarized in Supplementary Information S2, Table S7.

These results reflect observations from the regulation response pattern analyses, where each hub gene associates with a slightly different complement of genes. However, some hubs share genes in common, perhaps indicating expression in a common cell type. These results may also give some indication as to the cell type or biological processes associated with that hub gene, e.g., CD177 and MMP8 indicating neutrophil cell activity.

## Discussion

Using a two-tier gene screening process of rank distribution based on regression error and comparison with empirically-derived functional ontology lists derived from previously published literature, 48 significant genes were identified in this study. Three house-keeping genes for use in the study were additionally identified based on their invariant expression between disease test groups. Of these, 22 were upregulated in sepsis and 26 in SIRS. A gene interaction analysis based on these genes was conducted and 8 potent markers CD177, GPR84, FGF13, KLRK1, MYL9, PCOLCE2, TDRD9, and SLC16A3 were identified and selected for further investigation for their ability to stratify SIRS from sepsis and septic shock.

Gene interaction results for these 8 markers showed interaction relationships between other gene entities, which also showed dynamic regulation between different control and disease groups. The adult and pediatric control groups appear to exhibit a more balanced profile of negative and positive regulatory interactions between the hub and associated genes and may reflect a state of homeostasis. It is therefore not surprising to find some overlapped genes between pediatric healthy control and adult healthy control (see Supplementary Information 2, Table S6) although it is generally held that the immune responses of adults and children differ in some respects (110, 111). Fewer than nine overlapped genes were identified between these two non-disease groups and the majority of these genes were found to map to the KLRK1-associated immune response pathway.

In contrast, the disease group hub and associated gene interactions exhibit increased either positive or negative activities compared with normal controls. The adult and pediatric SIRS disease groups exhibited features in common i.e., shared interaction between the TDRD9 and PCOLCE2 hubs and the GPR84 and FGF13 hubs (the latter both directly and indirectly in adult SIRS). These interactions are replaced in pediatric resolved SIRS by direct interaction between the CD177 and GPR84 hubs. The adult sepsis group exhibited indirect interaction between the CD177, GPR84, and TDRD9 hubs whereas the pediatric sepsis group exhibited indirect interaction between CD177, GPR84 and TDRD9; direct interaction between the GPR84 and TDRD9 hubs, indirect interaction between TDRD9 and the FGF13 and PCOLCE2 hubs and also between the PCOLCE2 and SLC16A3 hubs. A similar interaction profile was observed in pediatric septic shock, except for the SLC16A3 interactions. This suggests commonalities in the core immune response between adults and children in sepsis and septic shock and this differs from the profile of interactions observed in SIRS. These results suggest that the immune pathways involved in SIRS and sepsis pathogenesis are different, even if the core hub entities controling those disease processes are shared. This could be taken to mean that either different response pathways are activated in the same cell type or that different cell types/sub types are involved in the response.

Comparing the overall interaction responses of the immune-related clusters CD177, GPR84, and KLRK1, stimulatory responses were observed between KLRK1 and its associated genes in adult sepsis, adult SIRS, pediatric SIRS and pediatric resolved SIRS. However, as this marker appears downregulated in the fold-change analyses compared with CD177 and GPR84 which are upregulated this would imply that the KLRK1 response is abrogated and that CD177/GPR84-responses are enhanced. Further detailed breakdown of these and the other hub associations may give an indication of some of the underlying pathological processes at work in the disease groups. CD177 (57, 58) and GPR84 (73, 74) are inflammatory markers and were found to be expressed at a high level in all disease groups compared to healthy controls. This suggests upregulation of immune processes associated with these markers in disease groups compared with healthy controls. This also further implies that sepsis and SIRS share elements of a common immune response blueprint, based around these two entities. These appear in opposite aspect in the overarching interactive pathways map (Figure 2). However, there are no significant variations in the overall expression level of these which could be used for stratifying sepsis from SIRS, septic shock, resolved-SIRS, or bacteraemia. There is a great deal of interactive action surrounding these two markers and these may fulfill oppositional roles in the primary disease processes. GPR84 stimulates more proinflammatory processes in adult SIRS, pediatric SIRS, pediatric resolved-SIRS, pediatric sepsis, and pediatric septic shock and would support the premise that these patients exhibit profiles consistent with an ongoing pro-inflammatory immune response. The precise nature and detail of these differ between each of the disease processes e.g., the interactions associated with GPR84 appear inhibitory in adult sepsis and stimulatory in SIRS and pediatric sepsis.

Central to this dynamic is the interplay between GPR84 and other entities e.g., CD177, FGF13, and PCOLCE2 which appears broadly inhibitory in nature in the overarching interaction map (Figure 2) and VSTM1(alternatively called SIRL-1), which is stimulatory. This latter entity is also being stimulated by DACH1. VSTM1 plays an extremely important role in the regulation of the oxidative burst in phagocytic cells (112, 113) including neutrophils and is involved in attenuating neutrophil extracellular trap (NET) formation (114, 115). It is also a key factor in development of the proinflammatory Th17 T-cell response (116). Thus, in the context of its antagonistic role to CD177, which when engaged with β2 integrins will impair neutrophil transmigration across endothelial barriers (57, 59, 60), this may perhaps imply differential control of two alternate differentiation and activation functions or pathways in neutrophils. CD177 appears to be subject to stimulatory mechanisms by HLA-DRA and GIMAP4, the former of which is likely expressed on other antigen presenting cells (APCs) and the latter by B and T-lymphocytes (117). The view currently perhaps represented by these interactions is of alternate promotion and/or competition between two different functional subtypes of neutrophils i.e., a proinflammatory/activated, trans-migratory subtype and an anti-inflammatory and/or differentiated subtype, perhaps involved in immune suppression and/or NET formation. This could also be interpreted as competition between two entirely different pro and anti-inflammatory cell types, as GPR84 can also be expressed by both neutrophils and monocytes/macrophages. It is not possible from this study to determine which cell type(s) are responsible for GPR84 expression and further study is required.

### CD177

As stated above, CD177 expression was observed to be highly elevated in adult and pediatric SIRS, sepsis, septic shock and resolved SIRS. It was found to associate with a variety of gene entities in the interaction analyses. These shared few commonalities between the different disease groups with the exception of DDAH2, RGL4, and ZDHHC19 in adult sepsis and pediatric septic shock. DDAH2 encodes a dimethylarginine dimethylaminohydrolase, which functions in nitric oxide (NO) generation, by regulating the cellular concentration of methylarginines responsible for inhibiting/regulating nitric oxide synthase activity (118). This entity has been associated previously with sepsis and may contribute to disease pathology through alterations in NO activity (118–120). In mice, knockouts (KOs) in DDAH2 result in increased mortality in a mouse model of sepsis (118), macrophage-specific DDAH2 KOs exhibit similar disease susceptibility to global KOs. Therefore, this gene entity is associated with the pro-inflammatory response and bacterial killing, as well as involvement in endothelial and blood pressure homeostasis. Its upregulation may perhaps also imply regulation of production of iNOS-regulated nitrogen reactive metabolites in sepsis (120). RGL4 overexpression leads to increases in the levels of the GTP-bound forms of Ral and the small GTPase Ras (121), leading to increased Ras activation and generation of morphologically transformed cells and disorganized cell growth. Additionally, ZDHHC19 is involved in palmitoylation of R-Ras (122), which leads to changes in its cellular functions, including increased viability and morphological alterations. Therefore, the activities of both these entities could be linked and might suggest regulation of cell morphological alterations. CD177 is a neutrophil specific gene and is variably expressed on different peripheral blood neutrophil populations (58). CD177^high^ expressing cells have been found previously in severe bacterial infections and sepsis (123, 124) and are considered to play a major role in disease pathology. Association of CD177 with other neutrophil associated genes e.g., LCN2 and ELANE was confirmed from similar entities analysis. Ras inhibits terminal neutrophil differentiation (125), thereby indefinitely extending their proliferative potential and strongly promotes the sensitivity of these cells to granulocyte-macrophage colony-stimulating factor (GM-CSF). These entities may perhaps therefore be implicated in accumulation of relatively undifferentiated, immature neutrophils in the peripheral blood as observed in sepsis and SIRS (126) and/or neutrophil NET formation (127). This latter process may be Ras mediated through the actions of RGL4 and ZDHHC19. Interestingly RGL4 is normally associated with KLRK1 in adult and pediatric healthy controls along with various other entities, implying an alternate role in normal healthy immune homeostasis.

CD177 also exhibited strong direct connections to other hub entities GPR84 and TDRD9 in adult and pediatric sepsis and to GPR84 alone in pediatric septic shock and resolved SIRS. It was not connected to any other hub entities in adult or pediatric SIRS. Similar profiles for GPR84 and CD177 were observed with the overlapped genes in sepsis (adult and pediatric–TDRD9) and in SIRS (adult compared with pediatric). This suggests that CD177-associated components of the response in adults and children are broadly similar but not identical in either disease condition.

In the similar entities analysis CD177 was linked with other classical neutrophil markers e.g., LCN2, ELANE and with others e.g., ARG1, ALPL, and CD24. It was also associated with ALPL in pediatric septic shock. ARG1 is a cytosolic arginase (128) and one of the factors expressed and released by myeloid suppressor cells (129). This depletes arginine in the cellular microenvironment leading to a number of immune-regulatory effects, including T-cell inhibition (130–132), limiting the availability of arginine for nitric oxide synthase, a key factor in NO production and regulation of other arginine-dependent biological processes (128). These myeloid suppressor cells have been implicated in the immunopathology of a number of diseases, but also in sepsis, both in humans and mouse models (130, 133). This combined with expression of DDAH2 as discussed above may suggest that these CD177 expressing cells may exhibit the characteristics of immature, anti-inflammatory myeloid (neutrophil) suppressor cells. This has been observed previously (131, 134, 135). Neutrophils and neutrophil NETs may play a key role in intravascular coagulation, which is a feature of sepsis (136, 137) and also be involved in immunomodulation (138–143).Therapies targeting these cells may be beneficial in sepsis (144, 145), although their role as disease driver or modulator remains unclear. As there are both pro and anti-inflammatory responses evident in sepsis, the pathway toward immunomodulatory therapies based on these observations, is still unclear (146).

ALPL is a tissue-non-specific form of alkaline phosphatase and is a membrane bound glycosylated enzyme not expressed in any particular tissue. Its function in disease processes in this study is unclear. We found it to be associated to adult SIRS (along with ZDHHC18, as opposed to ZDHHC19 found in the sepsis pathway association analysis). It has been associated with neutrophil activity previously in obesity (147) and may be associated with mitophagy of redundant mitochondria (148). Defective mitochondrial activity has also been previously documented in sepsis (149, 150). CD24 is considered to be predominantly a B cell marker (151), but it is also found on granulocytes and is implicated in early neutrophil responses to infection in sepsis-induced acute respiratory distress syndrome (152), along with other entities identified in this study—olfactomedin 4 (OLFM4), lipocalin 2 (LCN2), and bactericidal/permeability-increasing protein (BPI). These contribute further to the overall neutrophil-dominant profile of this hub and its associated entities.

Schaak et al. (20) observed an association of CD177 with MMP8, HP, and RETN in a large meta-analysis of previously-published sepsis microarray datasets and postulated this signature to be associated with activated neutrophils. These entities comprised the most preeminent, upregulated entities in this study, in addition to OLAH. These authors also noted significant expression heterogeneity between different individual patients, which may reflect differences in pathogenic disease origin. Our observations here would confirm some of those concluded earlier, from these previous studies. These observations, combined with the fact that CD177 appears to interact differently with other hub genes in either SIRS or sepsis, would imply perhaps activation of different pathological immune pathways but in a similar cell type (i.e., neutrophils). Different neutrophil subsets are becoming a keen focus of interest in disease pathogenesis (153) and further work is required to tease out these pathway relationships.

### GPR84

As with CD177, GPR84 showed increased interaction between the hubs, in disease states, compared with healthy controls. GPR84 is a G protein-coupled receptor for medium-chain fatty acids (74). It is expressed in neutrophils, monocytes and macrophages (75, 76) and can modulate pro-inflammatory TNFα mRNA expression in endotoxin-tolerant human monocytes when activated with its ligand decanoic acid (77). It is highly expressed on the surface of peripheral blood leukocytes and is responsible for IP3-Ca2+ signaling, mobilization of intracellular granules, chemotactic migration, as well as assembly of reactive oxygen species generating NADPH-oxidase. This latter function is relatively low in naïve neutrophils but is significantly increased by pre-treatment of neutrophils with TNFα (75) or GM-CSF (154). In both adult and pediatric SIRS there is a connection between GPR84 and FGF13, which in the pediatric group is indirectly through RETN and in adults directly and indirectly through CEACAM1 and CDADC1. This connection is maintained in pediatric resolved SIRS. RETN (resistin) is an immune-associated entity and a component of neutrophil exocytic granules (155) and inhibits essential neutrophil functions e.g., the oxidative burst (156). CEACAM1 is expressed on a variety of immune cells (42, 157), including neutrophils (158) and is a key receptor involved in binding of these cells to endothelial cells during the process of exvagination from the periphery into tissue locations (159, 160). CDADC1 (cytidine and dCMP deaminase domain containing 1) is reportedly a testis-specific protein involved in testis development (161), however it may play a role in regulating the cell cycle and proliferation (162, 163). Its role in disease processes in the context of this study is very unclear.

In adult sepsis there is indirect interaction between GPR84, CD177, and TDRD9 through EXOSC4 and with CD177 through NECAB1. The profile is somewhat similar in pediatric sepsis with connections between GPR84 and TDRD9 directly and indirectly through instead DDAH2 and with CD177 through C19orf59 and RGL4. These connections are maintained in pediatric septic shock, except mediated indirectly through ZDHHC19 (CD177) and PCYT1A (TDRD9). Entities ENTPD7, and EXOSC4 are shared between the adult and pediatric GPR84 connections. ZDHHC19 is linked to GPR84 in the pediatric interaction map as opposed to TDRD9 and CD177 in the adult interaction map. Thus, although the connections and entities are broadly similar the regulatory profile is significantly different and is inhibitory in the adults and stimulatory in children. Due to the key regulatory nature of GPR84, this would imply immune upregulation/inflammation in children associated with the GPR84 hub and suppression in adults. DDAH2, ZDHHC19 and RGL4 have been discussed previously (see above), entities ENTPD7, C19orf59, EXOSC4, and PCYT1A warrant further discussion.

ENTPD7 or ectonucleoside triphosphate diphosphohydrolase 7 is a purine-converting ectoenzyme which hydrolyses extracellular nucleoside triphosphates (UTP, GTP, and CTP) to nucleoside monophosphates as part of a purinergic signaling pathway. This is involved in inflammation and oxidative stress (164). Its role in cellular processes in this context are not known, however, it may be associated with protection from or response to oxidative stress (165), as hypoxia is a common feature of sepsis (166–169) and may have an effect on immune cell function (170). C19orf59 (or MCEMP1) was originally identified as a Mast cell-associated protein (171) and is a peripheral blood biomarker for patients with stroke (172–174). However, it also constitutes a component of neutrophil exosomes and myeloid suppressor cell exosomes (175). This factor perhaps plays a role in neutrophil pathophysiology, organ damage, or is associated with immune suppressor-cell activity in pediatric sepsis. EXOSC4 (exosome component 4) is also a component of exosomes, involved in 3′ → 5′ exoribonuclease activity (176), suggesting an association with degranulation activity. MYCL1 is the only entity exhibiting GRP84-associated stimulatory activity in adult sepsis. This entity is selectively expressed in dendritic cells (DCs), expression of which is controlled by IRF8 during DC development. MYCL1 expression is initiated in DC progenitors and maintained in mature DCs even in the presence of inflammatory signals such as granulocyte-macrophage colony-stimulating factor. Loss of L-Myc by DCs causes a significant decrease in *in vivo* T-cell priming during infection (177). This interaction may perhaps suggest that GPR84 reactivity is influenced by a MYCL1-associated process. PCYT1A (phosphate cytidylyltransferase 1, choline, alpha) is involved in the regulation of phosphatidylcholine biosynthesis. Its expression is somewhat ubiquitous, however is may play a role in some immune processes e.g., regulation of B cell progenitor fate in germinal centers.

The cell type involved in this GPR84 expression is not certain, as it can be expressed on neutrophils and other APCs e.g., monocytes/macrophages. In the similar entities analysis, it is variably linked with CD63, CD300A, and CD58, however there was no consensus between datasets. These biomarkers can also be expressed on several other cell types. Our hypothesis is that it is either expressed in a competitive fashion to the CD177-associated pathway in neutrophils or subsets of neutrophils, or in another APC type, functioning in a competitive fashion to CD177-expressing neutrophils. However, the activity of this marker could not be ascribed to a cell type with any degree of confidence.

### TDRD9

TDRD9 (tudor domain containing 9) also showed increased interaction between the hubs, in disease states, compared with healthy controls. Expression of this entity has been described in gametogenesis with expression in testis and thyroid (103–106), its association with immune cells or processes has not previously been reported. Its primary function is in transposon silencing and progression of spermatogenesis (107), through an ATPase activity and interaction with PIWI proteins. It potentially could play a broader role in DNA damage protection (108, 109). Whether or not this is a secondary function in immune-related processes is currently unknown.

In both adult and pediatric SIRS there is a connection with PCOLCE2, directly in the pediatric group and indirectly through OLAH in adults. This association is disconnected in pediatric resolved SIRS. No other entities were found to overlap between adult and pediatric groups. OLAH (oleoyl-ACP hydrolase), is another gene purported to have sex-linked tissue-restricted expression in placenta and testis and plays a role in fatty acid biosynthesis. Its expression in bone marrow-derived mononuclear cells isolated from patients with rheumatoid arthritis has been reported previously. Again, its functional role in the context of the SIRS disease process is as yet unexplained. Other placenta-related genes e.g., PLAC8 have also been reported in association with immune processes including sepsis, however wider tissue expression of this latter gene has already been mapped and its association with immune cells and function confirmed. Whether secondary functions exist for both TDRD9 and OLAH remains to be elucidated. In similar entities analysis it associated with many entities, including those shared between datasets CEACAM1, DACH1, and FLOT1. CEACAM1 has been discussed earlier, DACH1 is involved directly in regulation of cell cycle regulators in cells of myeloid origin (178) and FLOT1 involved in vesicle trafficking and cell morphology and its expression documented previously in immune cells (179–181).

TDRD9 appears to play a central and dominant role in sepsis, particularly the pediatric data set. As described above, there was association of TDRD9 with CD177 and GPR84 in adult and pediatric sepsis. In addition, there was association with FGF13 through BST1 and PCOLCE2 through SH3GLB1 in pediatric sepsis. Connection with CD177 is lost in pediatric septic shock. SH3GLB1 (SH3 domain containing GRB2 like, endophilin B1, also called Bif-1) is a proapoptotic member of the Bcl-2 family, Bcl-2-associated X protein (Bax) and may be involved in regulating apoptotic signaling pathways and in maintaining mitochondrial morphology. This may play a role in autophagy-mediated pathogen killing (182, 183). The overarching impressions from the TDRD9-associated activities are of cell proliferation, adhesion and phagocytosis/autophagy, perhaps involved in pathogen clearance/killing (184).

### FGF13

The association of FGF13 with GPR84 in pediatric and adult SIRS has been discussed. This association is lost in pediatric resolved SIRS. This entity also shows association with RETN, LCN2, and crucially OLFM4 in pediatric SIRS and C19orf59 in adult SIRS. OLFM4 (olfactomedin 4) is an antiapoptotic factor which plays a role in several cellular and immune functions and is associated with a subset of neutrophils (61), distinct from CD177 expressing neutrophils (185). OLFM4 expressing neutrophils have been suggested to be the pathogenic cell type in a murine model of sepsis (186) and in pediatric septic shock (187) and has been trialed along with other neutrophil genes as candidate biomarkers in sepsis (188). FGF13 is also associated with TDRD9 in pediatric sepsis, as discussed previously and no other hub entities in adult sepsis. It is also associated with TDRD9 in pediatric septic shock and indirectly with PCOLCE2 through WIPI and ASPH. WIPI is an autophagy-associated entity, which is implicated in neutrophil differentiation from immature precursors. An inhibitory interaction between this and FGF13, would imply an effort to prevent neutrophil differentiation in pediatric septic shock. ASPH (aspartate beta-hydroxylase) is involved in ion and small molecule transport pathways, its role in this disease process context is unclear. Similar entities analysis did not provide any clear identification of this entity with a specific cell type, due to lack of clarity in association with other cell-type specific gene markers (Supplementary Information S2, Table S7). Interestingly it does appear to associate with a large number of CD markers, including CD1A, CD1B, and CD1C, among others, which might support a function of presentation of lipid antigens to NKT cells. However, based on the ANN evidence, it may be that FGF13 is neutrophil associated. It sits at the center of the overarching interaction map (Figure 2) and is the target for negative interactions with CD177, MMP8, and RETN, which all associate together in the similar entities analysis. This could be taken to imply competitive interaction between two neutrophil cell types, one OLFM4 expressing (186, 189, 190) and the other CD177 expressing (191), perhaps associated with different immune functions. This requires further investigation.

### PCOLCE2

PCOLCE2 interactions have been described in the context of other hub entities previously. It appears to have a considerable dependency on interactions with others, particularly TDRD9. It interacts with this entity only in adult and pediatric SIRS. It becomes disconnected from TDRD9 in pediatric resolved SIRS. It does not interact with any other entities in adult sepsis. It associates directly with TDRD9 and indirectly with SLC16A3 in pediatric sepsis, through MTF1, CD82, and G6PD. It is associated with TDRD9 and FGF13 in pediatric septic shock, as discussed previously. PCOLCE2 also critically interacts with another entity CMTM4 (192), which is an enhancer of PD-L1 activity. This could play a major role in immune suppression of T-cell activity. MTF1 (metal regulatory transcription factor 1), plays a primary role in metal ion homeostasis and processing, but also a role in fatty acid and lipid metabolism. It may have a function in ion and metabolic homeostasis (193, 194), during the disease inflammatory process. It's association with G6PD (glucose 6-phosphate dehydrogenase) and various other factors including CA4 (carbonic anhydrase 4) and HK3 (hexokinase 3) would also imply a central role in metabolic homeostasis sensing or regulation. This may be a secondary effect in the disease process, due to metabolic perturbation and hypoxia seen in both these disease conditions. Its primary role in disease pathology is unclear. CD82 is a cell surface tetraspannin (195, 196), expressed on a variety of leukocyte cells including T cells (197), is downregulated in infection and correlates with increased leukocyte motility. Similar entities analysis gave a similar profile to that for TDRD9 and provided little useful information as to the cells of origin, although it provides additional evidence of the close functional link between TDRD9, PCOLCE2, and also SLC16A3. The functions managed by these hub genes may be intimately connected.

### SLC16A3

SLC16A3 [solute carrier family 16 (monocarboxylic acid transporters), member 3], showed no increased interaction between the hubs in any of the disease states except pediatric sepsis compared with healthy controls. This entity plays a role in transport of monocarboxylates including lactic acid and pyruvate. Lactate is postulated to increase PD-L1 expression on monocytes and macrophages which could contribute to immunosuppression (198). It associates with CD82 and MTF1 (see above), in both pediatric SIRS and sepsis (see above) and DENND3 and MBD6 in pediatric sepsis and septic shock. It is also associated with FOSL2 in pediatric septic shock and LILRB2 and ITGAM in pediatric resolved SIRS. It is associated with FUT7 and AMPD3 in adult SIRS and sepsis. This entity therefore shows some overlap in activity between SIRS and sepsis, although again the mechanisms at play in adults and children is different. DENND3 (DENN domain containing 3) is the exchange factor for the small GTPase Rab12 and is involved in macro-autophagy (199–202), induced in response to cellular starvation. MBD6 (methyl-CpG binding domain protein 6) is involved in intracellular protein metabolism (203, 204) and may play a role in innate system pathogen killing (205). Its function therefore may be linked to that of DENND3. AMPD3 (adenosine monophosphate deaminase 3) is also involved in energy metabolism and may also play a role in microbial interactions with the host (206).

FOSL2 expression is regulated by TGF-β1 and is a key transcriptional regulator which plays a role in invariant natural killer T-cell (iNKT) development (207). Alteration of expression of this marker would suggest a role of these cells in pediatric sepsis. LILRB2 is involved in immunosuppressive responses (208–210) and may function in down-regulation of immune responses in pediatric resolved SIRS. ITGAM (CD11b) is a component of the receptor for platelet factor 4 (PF4), but also functions in immune signaling connections with SLC16A3. This latter entity is a general myeloid cell marker, including myeloid suppressor cells, which have been implicated in sepsis and other diseases (130, 133, 211–215). These associations would suggest that this hub entity may play a key role in immunosuppressive cell functions (132), as its effect on many entities in the overarching pathways map is inhibitory (Figure 2). FUT7 [α1,3-fucosyltransferase (Slex)] is involved in leukocyte/endothelial adhesion (216) in response to IL1β stimulation (217) and its involvement implies activation and binding of cells as part of the functionality associated with this hub entity in adults. CD58, a ligand of the T lymphocyte CD2 protein, which functions in adhesion and activation of T lymphocytes (218–220) was found to be associated with SLC16A3 by similar entities analysis and this may be of significance in that engagement of this key receptor with CD2 is predominant in chronic antigen stimulation, which may be an important feature of sepsis, particularly with regard to natural killer cell (NK) cell dysfunction (221). The predominant theme of this hub and associated entities is therefore effector/suppressor cell function and associated T, NK, or invariant natural killer cell (iNKT) cell activity in sepsis and a somewhat different pathological pathway or cell type associated with this marker in pediatric compared with adult SIRS and sepsis.

### KLRK1

KLRK1 (killer cell lectin like receptor K1, also called NKG2D) is a transmembrane protein involved in natural killer cell (NK cell) and CD8 T-cell activation (222–224). It has been shown to also regulate T-cell independent B1-subtype B cells and antibody production in response to bacterial infection (82). NKG2D-deficient mice produce significantly less antigen-specific IgM antibodies upon immunization with T cell-independent antigens, and they are more susceptible to Gram-negative sepsis. This is therefore a crucial key entity responsible for cell activation in innate and adaptive immune responses and plays an important role in protection from infection and development of sepsis.

Expression of this entity was reduced in pediatric SIRs and sepsis and this was maintained in septic shock and resolved SIRS. Its expression remained relatively unchanged in adult SIRS and sepsis. It is interesting that this marker is downregulated in these disease conditions and alongside many of the associated entities, appears to be inhibited. KLRK1 did not associate with any other hubs in any of the control or disease states. It showed association with CD2, IL2RB, and IL7R in adult SIRS and KLRG1 and TGFBR3 in adult sepsis, among others. It showed interaction with CD28 and TLR7 in pediatric SIRS and CD8A, KLRC3, GIMAP5, and GIMAP6 in resolved-SIRS. There is connection between KLRK1 and CD2, NLRC3 (a NOD-like receptor involved in innate immunity), CD247 (CD3-ξ chain), CD96 and other markers of T and NK function. Association with many of these markers was confirmed using similar entities analysis (Supplementary Information S2, Table S7) e.g., CD2, CD8A, IL2RB, ITK, NLRC3, and also GIMAP4, etc. Many of these entities are T-cell associated and this would imply a primary associative interaction with T-cell derived actions. Although generally T-cell function is also impaired in sepsis little is known about the action of γδ or invariant NKT cells in disease processes, or mucosal associated invariant T cells (MAITs) and intraepithelial lymphocytes (IELs) (225).

GIMAP4 sits at the fulcrum of the overarching interaction map and is a stimulatory effector of several key entities, including PCOLCE2, FGF13, and CD177. Expression of this entity is prevalent in B and T-cells (117) and particularly mature T cells (226). It exhibits differential expression in cells of the T helper (T_h_) subtype (227), affecting expression of key cytokines including interferon-γ (228). This observation could be interpreted therefore, as T_h_ cells being the positive stimulatory driver behind these latter entities activities. The other entities in the complex frame centered around them in Figure 2 interaction map, appear to be seeking to counteract and oppose this activity i.e., there are a lot of directed inhibitory activities directed against them by other regulatory factors in pediatric sepsis e.g., BMX (229) and VSTM1 (116) etc. KLRK1 showed increased interaction with a variety of entities including BCL11B in pediatric sepsis.

In addition, KLRK1 appears negatively influenced by kinases ITK [inducible T-cell kinase (230)] and TXK. These are involved in CD4, CD8, and iNKT cell development (231–239). ITK functions to regulate mature T-cell development and differentiation, but also acts as an inhibitor of NKG2D-initiated granule-mediated killing in NK cells (83, 240). This implies a central role in NK cell down-regulation. NK cell function is impaired in sepsis (241–244) most likely due to chronic antigen overstimulation (221). Therefore, the overarching and complex emerging picture is of NK-cell function suppression, perhaps driven by (CD4) T-cell and/or APC activity. This is more profound in the pediatric data set than the adult. NK cell dysfunction has become a focus of research interest in recent reports and may be associated with chronic antigenic stimulation and exhaustion (18, 221, 241, 243). This may be due to defective receptor-mediated effector responses (242), and the observations presented here would support that hypothesis.

### MYL9

MYL9 (myosin light chain 9) is primarily a myosin light chain that may regulate muscle contraction by modulating the ATPase activity of myosin heads. However, various isoforms of this exist. The inducible form MLC-2C plays a role in monocyte/macrophage lineage development and platelet production (85–87). In this study MYL9 associates with a large number of other genes with platelet-related functions e.g., CMTM5, ITGA2B, ITGB3, TREML1, and SELP (245), which may perhaps suggest its role in this context is as regulator of platelet production.

This hub gene exhibited the greatest conservation of associated entities, compared with other hub entities, across all the control and disease interaction map analyses. However, it also did not associate with any other hubs in any of the control or disease states. In the pediatric control it associated with a number of entities including SELP, CMTM5, and TGF1l1, in pediatric SIRS with ITGB3 and ITGA2B, TREML1, and CMTM5. All of these are associated with MYL9 in pediatric sepsis and in pediatric septic shock, except for CMTM5 in the latter. CMTM5, SELP, and TREML1 were also observed in pediatric resolved SIRS, in addition to ALOX12 (arachidonate 12-lipoxygenase, 12S type), which was also seen in pediatric sepsis. This latter entity and its reaction products have been shown to regulate platelet function (246). The profile of MYL9 associated gene interactions for adult sepsis is quite different than that for pediatric sepsis, with association instead with platelet membrane glycoproteins GP5 and GP6, PLOD2, COL6A3, and PROS1. MYL9 associates with TREML1 in adult controls and other integrin subunits ITGA3 and ITGB3BP. It interacts with TREML1, CMTM5, TGFB1l1, and ITGA2B in adult SIRS, among others and TGFB1l1 only in adult sepsis. The interactions are intermediate in adult SIRS and predominantly pro-stimulatory in adult sepsis.

SELP (p-selectin) has been implicated in sepsis for some considerable time and may contribute to neutrophil recruitment and NET formation (247–250). Ascorbic acid treatment is in focus as a potential therapy directed toward these cell-to-cell interactions (251, 252). CMTM5 has been implicated in platelet aspirin responses through studies in cardiovascular disease (253, 254). ITGB3 (integrin subunit beta 3) and ITGA2B (integrin subunit alpha 2 beta; both component chains of the heteromeric, platelet-specific integrin αIIbβ3), have also been the focus of intense interest in sepsis (255–258). TREML1 is an α-granule-localized platelet factor, which functions in aggregation, bleeding, and inflammation (259–261), of which soluble forms are of interest in disease control (262, 263). TGFB1I1 (Hic-5) is a transforming growth factor β1 (TGF-β1) -induced matrix protein, involved in matrix formation and cell adhesion (264–273). To our knowledge its association with platelet gene transcript profiles has not previously been reported.

The profile of interactions for adult sepsis is quite different than that for pediatric sepsis, with association instead with platelet membrane glycoproteins GP5 and GP6, PLOD2, COL6A3 and PROS1, ITGA3, and ITGB3BP (integrin subunit beta 3 binding protein), inferring activation down a different pathway. GP5 (human platelet glycoprotein V) is a part of the Ib-V-IX system of surface glycoproteins that comprise the receptor for von Willebrand factor and mediate the adhesion of platelets to injured vascular surfaces in the arterial circulation (274–278). GP6 forms a complex with the Fc receptor gamma-chain to initiate the platelet activation signaling cascade upon collagen binding (279–284) and may be associated with an increased risk of platelet-related disorders including ischemic stroke. PLOD2 (procollagen-lysine,2-oxoglutarate 5-dioxygenase 2) catalyzes the hydroxylation of lysyl residues in collagen-like peptides and COL6A3 (collagen type VI alpha 3 chain) is a general structural matrix protein and is fairly ubiquitously expressed. However, it is differentially regulated during hypoxia (285, 286) and may alter function during inflammatory stress. PROS1 encodes a vitamin K-dependent plasma protein that functions as a cofactor for the anticoagulant protease, activated protein C (APC), which functions to inhibit blood coagulation (287–291) and is vitamin K dependant (292). Association of many of the above described entities with MYL9 was confirmed using similar entities analysis (Supplementary Information S2, Table S7).

Many of the above entities are components of platelet α-granules (293). Thus, platelet activity, aggregation and blood clotting feature as a dominant theme within the MYL9 associated entities. The pathways of activation appear different between adults and children. Thus, the overarching view across the control and disease interaction maps, is of perhaps a state of platelet homeostatic balance in healthy controls then a shift to a predominantly inhibitory process between these gene elements in pediatric SIRS, which then alters to a stimulatory aspect in resolved SIRS. The interactions are profoundly stimulatory in sepsis and septic shock. This process might in part be driven by increased thrombopoietin levels in the blood of sepsis patients (294, 295) and has previously been acknowledged to be a significant contributing factor in organ failure (296).

### Significant Pathways

Many of the above pathway observations were also confirmed from the results of the pathway analysis. Entities in the blood coagulation pathway (P00011) were found to be overlapped in SIRS, sepsis, and septic shock. These pathway reactions are perhaps revealed in the profile of entities associated with MYL9. The integrin signaling pathway (P00034) was also found to be represented in all disease groups and the healthy controls. Tissue injury caused by infection and also by an inappropriate host response, can lead to multiple dysfunctions during sepsis progression.

## Summary

A two-tier gene screening using ANNs was conducted to identify a panel of markers and their relationships related to sepsis and SIRS. On the first-tier screening process, a panel of 48 candidate markers and 3 housekeeping genes were identified. Amongst these 48 candidate markers, 26 showed a consistent upregulation response in adult SIRS and the remaining 22 were upregulated in adult sepsis. The interaction between these genes were then analyzed in the second-tier screening process. Eight key hub genes were identified. Further investigation on pathogenesis activities of these hub genes in stratifying sepsis from SIRS, septic shock, resolved SIRS and bacteraemia were conducted.

Our findings are summarized below:

- CD177 can be taken to imply a substantial infiltration of CD177 positive neutrophils in both SIRS and sepsis
- GPR84 appears to oppose and provide an inhibitory control for CD177 and this may be taken to mean diametrically opposing and competing pathways, either in the same cell type or subtypes, or in two different cell types. The cell-type assignation is unknown, and this requires further investigation
- TDRD9 may be involved in regulation of cell proliferation, adhesion and phagocytosis/autophagy, perhaps involved in pathogen clearance/killing. The cell type assignation is also unknown and requires further investigation
- FGF13 may be neutrophil-associated and shows some linkage with hub genes TDRD9 and PCOLCE2
- The pathogenic role or cell association of PCOLCE2 and SLC16A3 are unclear, but these may function in metabolic processes, perhaps associated with hypoxia and carboxylic acid metabolism and are closely linked to each other and TDRD9. They may also be linked to immunosuppressive cell activities, however this requires further investigation
- KLRK1 is most likely associated with NK cell activity and its reduction in expression can be taken to mean loss or down-regulation of NK cells/markers from the periphery. This could be due to inhibition, cell death or egress from the periphery
- KLRK1 inhibition may be actioned through ITK
- This process appears to be inhibited/driven by T-cells and/or APCs, which also appear to promote the activities of other key hub genes such as CD177, FGF13, and PCOLCE2
- The activity of MYL9 could be ascribed to platelet activation and blood clotting cascades with reasonable confidence
- The activity surrounding MYL9 is a key differentiator between SIRS and sepsis in both adults and children, with apparent inhibition of these activities/pathways in SIRS and activation in sepsis
- This is consistent with the observation of intravascular coagulation being a key feature of disease pathology in sepsis and a major cause of observed organ damage.

While the hub genes showed significant fold increases over controls, they did not show sufficient fold-change differences between SIRS and sepsis and lack discriminatory power to differentiate these two conditions. Although some of these hubs and associated gene interactions cannot yet be easily described in the context of this study, they may provide the basis for further investigation into gene regulatory networks and pathways and their specific involvement in disease development and progression. They may also provide a new source of other biomarkers for prospective investigations in differential diagnosis. A better understanding of the disease processes underpinning each condition, could also pave the way for improvements in patient management and care. The interaction analysis has provided additional information as to the likely source of a few of the hub markers e.g., CD177, FGF13, KLRK1, and MYL9, which could be ascribed to a cell type with reasonable confidence i.e., neutrophils, NK cells, and platelets. These cell types have been implicated previously in the immunopathology of sepsis and this may pave the way for new or improved targeted therapies. The remaining hub genes GPR84, TDRD9, PCOLCE2, and SLC16A3 could not be assigned to a cell type with the same degree of confidence and their cell-associated expression and role in underlying disease immunopathology requires additional study. Further work is in progress to investigate these biomarkers and associated pathways, to provide clarification on the likely basis of cell type-associated expression and identify diagnostically useful biomarkers.

## Data Availability Statement

Publicly available datasets were analyzed in this study. These data are available in the ArrayExpress repository using the following accession numbers: E-GEOD-9960, E-GEOD-28750, E-GEOD-13904, E-GEOD-6269.

## Ethics Statement

Ethical review and approval was not required for the study on human participants in accordance with the local legislation and institutional requirements. Written informed consent for participation was not required for this study in accordance with the national legislation and the institutional requirements.

## Author Contributions

DT and KK conducted the data analysis. DT, KK, TS, and GB wrote and edited the paper.

### Conflict of Interest

The authors declare that the research was conducted in the absence of any commercial or financial relationships that could be construed as a potential conflict of interest.

## Supplementary Material

Supplementary Information S1 **Figure S1:** Rank distribution of all 54675 probe sets in the study, based on regression errors. **Figure S2:** Boxplot depiction of median expression values in all control and disease groups. **Figure S3:** Fold change expression differences in all disease groups relative to healthy controls. **Figure S4:** Median expression of core hub markers across all healthy control and disease groups. **Figure S5:** Gene feature representation in PANTHER functional pathways across all control and disease groups.

Supplementary Information S2 **Table S1:** Product of *p*-values using the rank order from ANN data modeling and Student *T*-tests, computed using the BH-FDR. **Table S2:** Fold-change expression values of the key hub markers across all healthy control and disease groups. **Table S3:** Predictive error values of the key hub markers across all healthy control and disease groups. **Table S4:** Number of overlapped and non-overlapped gene shared between the key hub markers across all healthy control and disease groups. **Table S5:** Pathway association using PANTHER functional pathways analysis across the adult healthy control, SIRS, and sepsis groups. **Table S6:** Overlapped genes shared between the key hub markers across all healthy control and disease groups. **Table S7:** Gene features associated with the key hub genes and shared between the data sets used in the study, identified using GeneSpring 12.5^TM^ similar entities analysis.

Supplementary Information S3 **Table S3.1:** In the similar entities Select gene biomarker features used in the similar entities analysis.

Supplementary Information S4 Description and discussion of gene interaction patterns in pediatric gram-negative and gram-positive bacteraemia.

Supplementary Information S5 **Figure S5.1:** Hub Gene Entity Interaction Map for Pediatric Gram-Positive Bacteraemia. Figure S5.2. Hub Gene Entity Interaction Map for Pediatric Gram-Negative Bacteraemia.

Supplementary Information S6 **Table S6.1:** ANOVA results for dataset E-GEO 6269, ranked on significance (*p*-value from lowest to highest). **Table S6.2:** Results of similar entities analysis for CD177 using dataset E-GEO 6269. **Table S6.3:** Results of similar entities analysis for FGF13 using dataset E-GEO 6269. **Table S6.4:** Results of similar entities analysis for GPR84 using dataset E-GEO 6269. **Table S6.5:** Results of similar entities analysis for KLRK1 using dataset E-GEO 6269. Table S6.6. Results of similar entities analysis for MYL9 using dataset E-GEO 6269. **Table S6.7:** Results of similar entities analysis for PCOLCE2 using dataset E-GEO 6269. **Table S6.8:** Results of similar entities analysis for SLC16A3 using dataset E-GEO 6269. **Table S6.9:** Results of similar entities analysis for TDRD9 using dataset E-GEO 6269.

Supplementary Information S7 **Table S7.1:** ANOVA results for dataset E-GEO 28750, ranked on significance (*p*-value from lowest to highest). **Table S7.2:** Results of similar entities analysis for CD177 using dataset E-GEO 28750. **Table S7.3:** Results of similar entities analysis for FGF13 using dataset E-GEO 28750. **Table S7.4:** Results of similar entities analysis for GPR84 using dataset E-GEO 28750. **Table S7.5:** Results of similar entities analysis for KLRK1 using dataset E-GEO 28750. **Table S7.6:** Results of similar entities analysis for MYL9 using dataset E E-GEO 28750. **Table S7.7:** Results of similar entities analysis for PCOLCE2 using dataset E-GEO 28750. **Table S7.8:** Results of similar entities analysis for SLC16A3 using dataset E-GEO 28750. **Table S7.9:** Results of similar entities analysis for TDRD9 using dataset E-GEO 28750.

Supplementary Information S8 **Table S8.1:** ANOVA results for dataset E-GEO 13904, ranked on significance (*p*-value from lowest to highest). **Table S8.2:** Results of similar entities analysis for CD177 using dataset E-GEO 13904. **Table S8.3:** Results of similar entities analysis for FGF13 using dataset E-GEO 13904. **Table S8.4:** Results of similar entities analysis for GPR84 using dataset E-GEO 13904. **Table S8.5:** Results of similar entities analysis for KLRK1 using dataset E-GEO 13904. **Table S8.6:** Results of similar entities analysis for MYL9 using dataset E-GEO 13904. **Table S8.7:** Results of similar entities analysis for PCOLCE2 using dataset E-GEO 13904. **Table S8.8:** Results of similar entities analysis for SLC16A3 using dataset E-GEO 13904. **Table S8.9:** Results of similar entities analysis for TDRD9 using dataset E-GEO 13904.

Supplementary Information S9 **Table S9.1:** ANOVA results for dataset E-GEO 9960, ranked on significance (*p*-value from lowest to highest). **Table S9.2:** Results of similar entities analysis for CD177 using dataset E-GEO 9960. **Table S9.3:** Results of similar entities analysis for FGF13 using dataset E-GEO 9960. **Table S9.4:** Results of similar entities analysis for GPR84 using dataset E-GEO 9960. **Table S9.5:** Results of similar entities analysis for KLRK1 using dataset E-GEO 9960. **Table S9.6:** Results of similar entities analysis for MYL9 using dataset E-GEO 9960. **Table S9.7:** Results of similar entities analysis for PCOLCE2 using dataset E-GEO 9960. **Table S9.8:** Results of similar entities analysis for SLC16A3 using dataset E-GEO 9960. **Table S9.9:** Results of similar entities analysis for TDRD9 using dataset E-GEO 9960.
