# Supplementary material for: Development of a Bioinformatics Framework for Identification and Validation of Genomic Biomarkers and Key Immunopathology Processes and Controllers in Infectious and Non-infectious Severe Inflammatory Response Syndrome
Source: Front Immunol. 2020 Mar 31;11:380. doi: 10.3389/fimmu.2020.00380 (PMC7147506; doi:10.3389/fimmu.2020.00380)
Supplement: Supplementary Information S1 — Figure S1: Rank distribution of all 54675 probe sets in the study, based on regression errors. Figure S2: Boxplot depiction of median expression values in all control and disease groups. Figure S3: Fold change expression differences in all disease groups relative to healthy controls. Figure S4: Median expression of core hub markers across all healthy control and disease groups. Figure S5: Gene feature representation in PANTHER functional pathways across all control and disease groups. [file Table_1.DOCX]

**Supplementary Information S1**

**Figure S1**

**
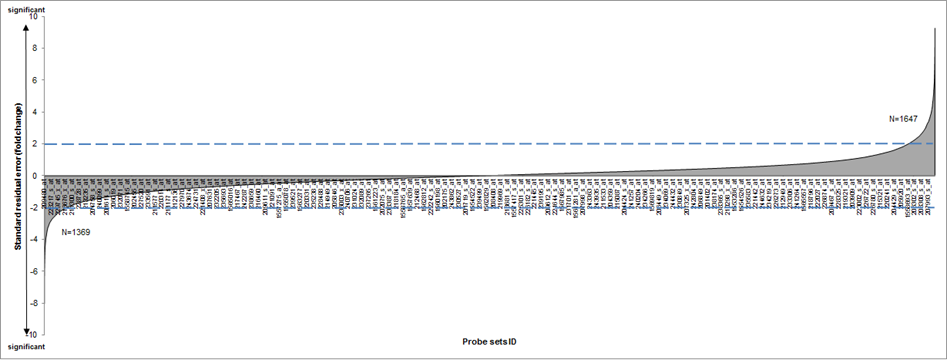
**

**Figure S2**

**
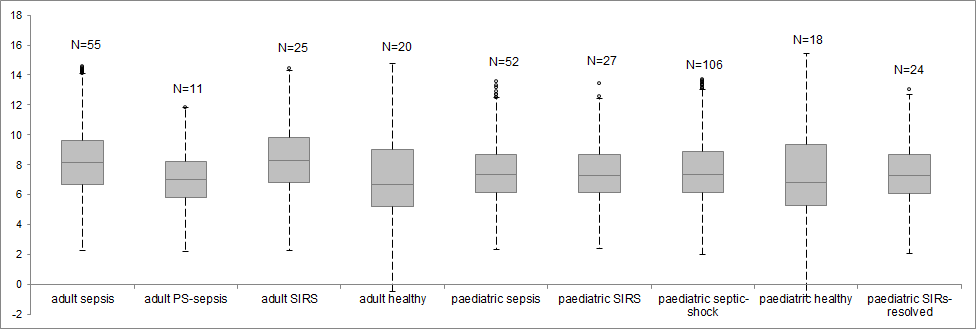
**

**Figure S3**

**
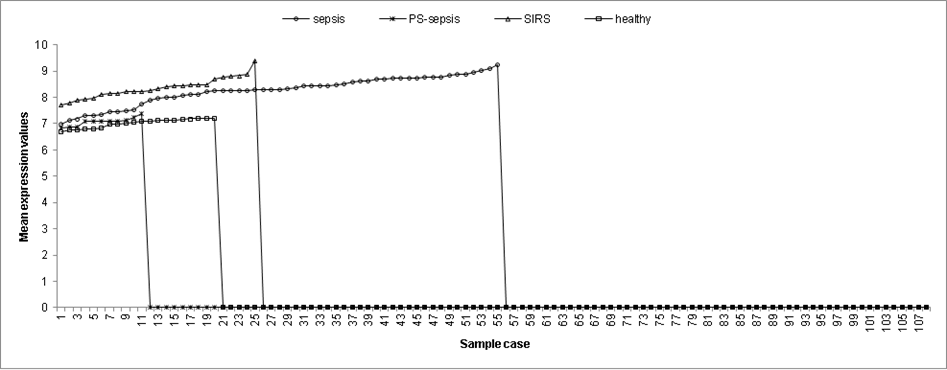
**

**Figure S4**

**
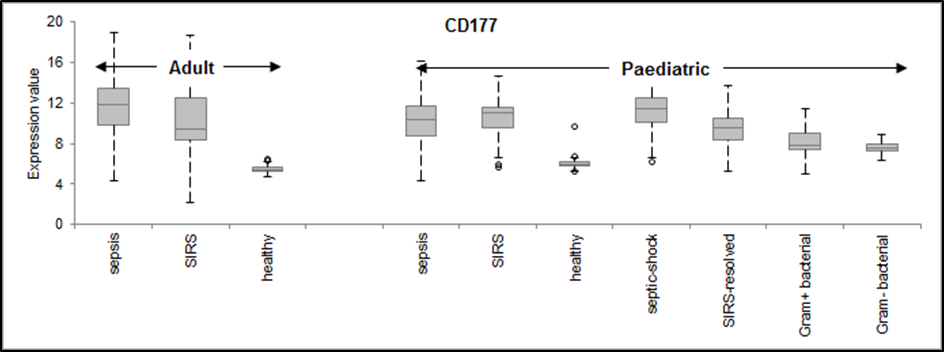
**

**(a) CD177**

**
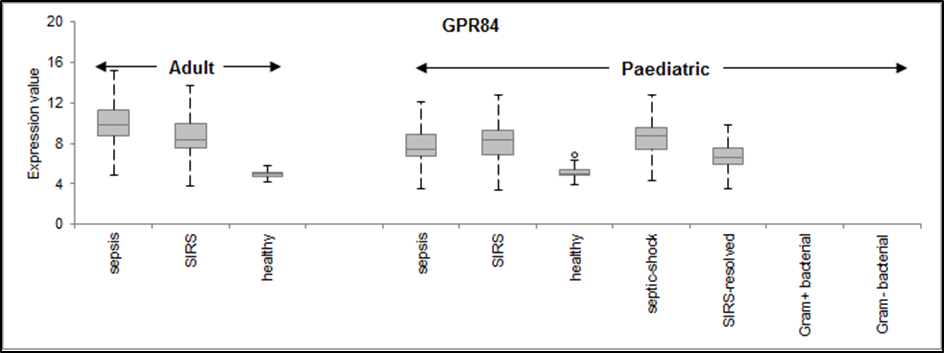
**

**(b) GPR84**

**
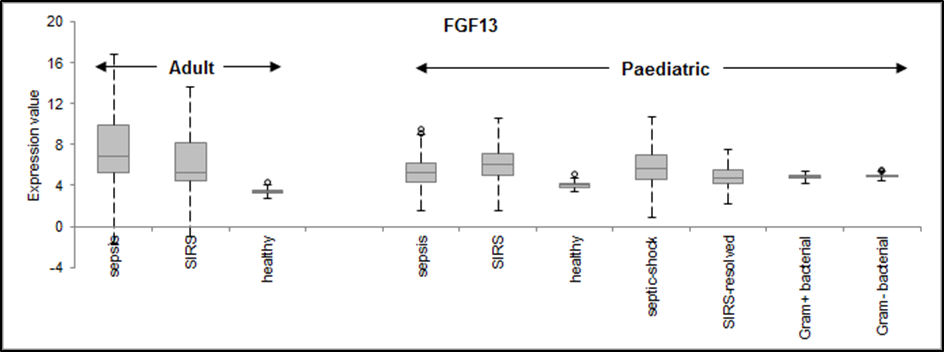
**

**(c) FGF13**

**
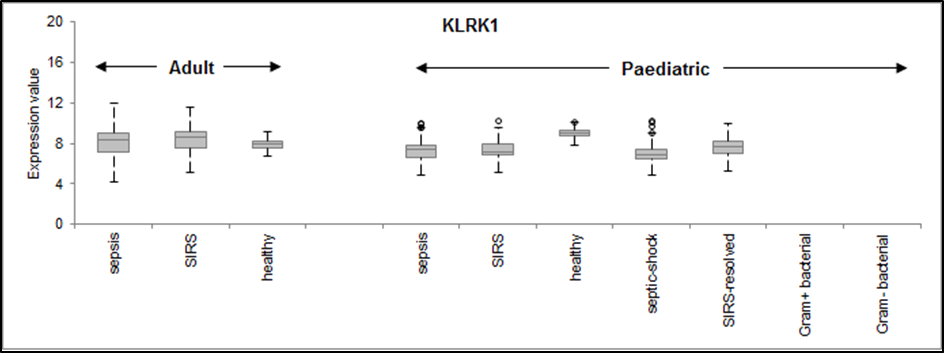
**

**(d) KLRK1**

**
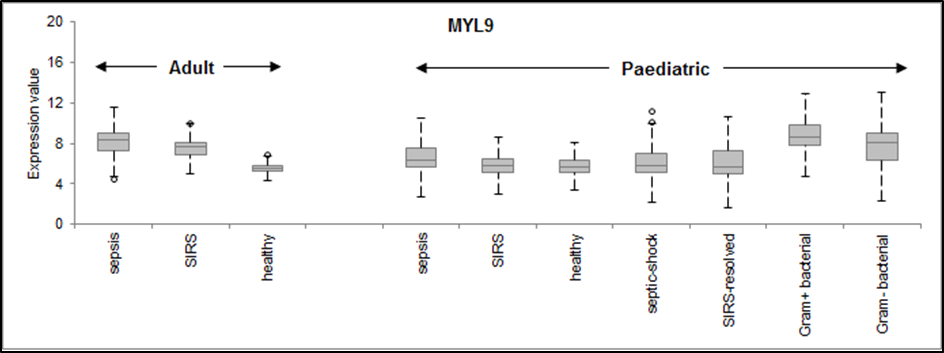
**

**(e) MYL9**

**
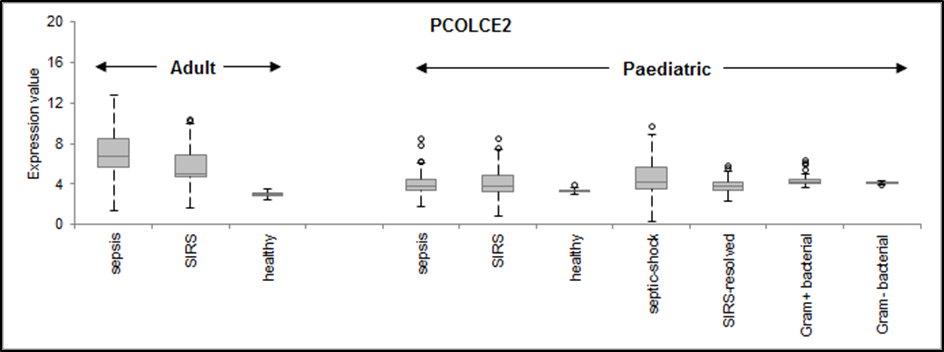
**

**(f) PCOLCE2**

**
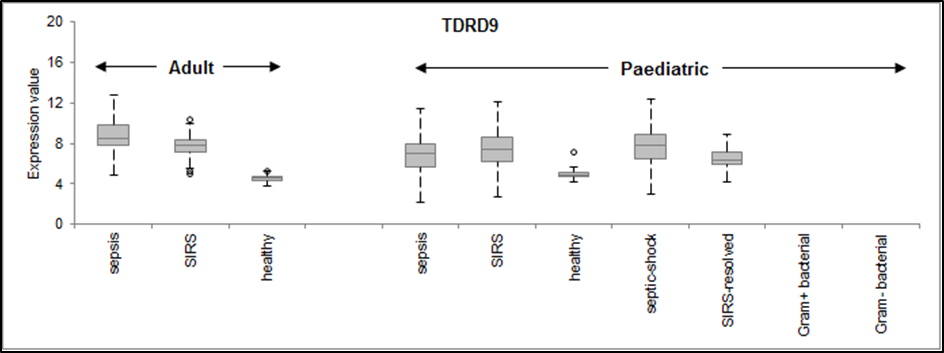
**

**(g) TDRD9**

**
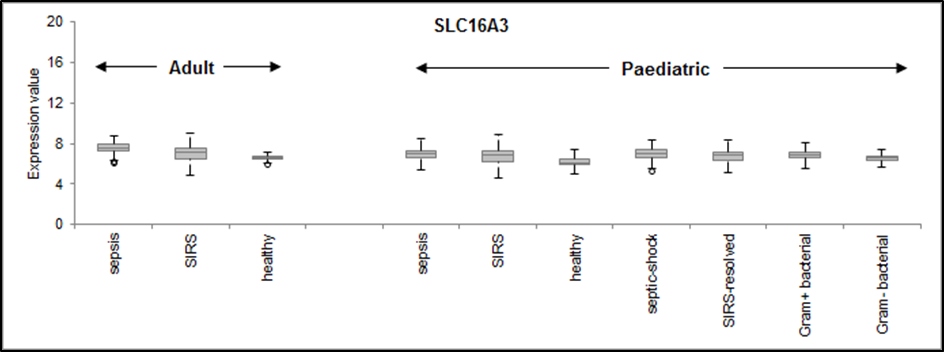
**

**(h) SLC16A3**

**Figure S5**

**
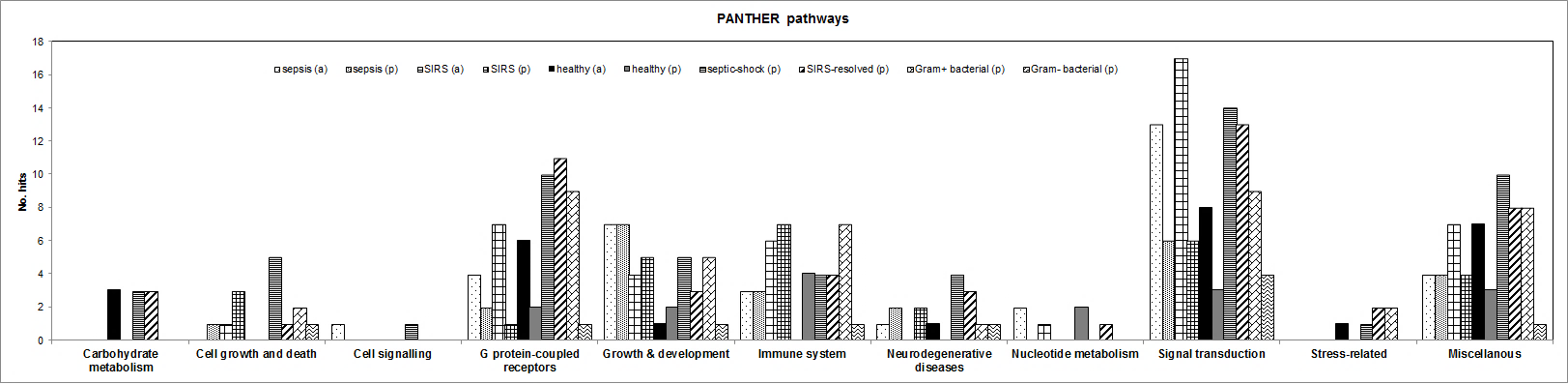
**

**Feature representation in PANTHER functional pathways.**
