# Supplementary material for: Development of a Bioinformatics Framework for Identification and Validation of Genomic Biomarkers and Key Immunopathology Processes and Controllers in Infectious and Non-infectious Severe Inflammatory Response Syndrome
Source: Front Immunol. 2020 Mar 31;11:380. doi: 10.3389/fimmu.2020.00380 (PMC7147506; doi:10.3389/fimmu.2020.00380)
Supplement: Supplementary Information S2 — Table S1: Product of p-values using the rank order from ANN data modeling and Student T-tests, computed using the BH-FDR. Table S2: Fold-change expression values of the key hub markers across all healthy control and disease groups. Table S3: Predictive error values of the key hub markers across all healthy control and disease groups. Table S4: Number of overlapped and non-overlapped gene shared between the key hub markers across all healthy control and disease groups. Table S5: Pathway association using PANTHER functional pathways analysis across the adult healthy control, SIRS, and sepsis groups. Table S6: Overlapped genes shared between the key hub markers across all healthy control and disease groups. Table S7: Gene features associated with the key hub genes and shared between the data sets used in the study, identified using GeneSpring 12.5TM similar entities analysis. [file Table_2.DOCX]

**Supplementary Information S2**

**Table S1**

| **Gene** | **Entrez ID** | **Probe Set ID** | **Cytoband** | **Differential**  **Regulation**  **Disease vs**  **Control**  **(upregulation)** | **p-value*** |
| --- | --- | --- | --- | --- | --- |
| **HP** | 3240 | 206697_s_at | 16q22.2 | Sepsis | 1.99E-03 |
| **METTL7B** | 196410 | 227055_at | 12q13.2 | Sepsis | 1.03E-02 |
| **TDRD9** | 122402 | 228285_at | 14q32.33 | Sepsis | 1.05E-02 |
| **PCOLCE2** | 26577 | 219295_s_at | 3q21-q24 | Sepsis | 1.28E-02 |
| **VSTM1** | 284415 | 235818_at | 19q13.42 | Sepsis | 1.37E-02 |
| **BPI** | 671 | 205557_at | 20q11.23 | Sepsis | 3.77E-02 |
| **DACH1** | 1602 | 205471_s_at, 228915_at, 205472_s_at | 13q22 | Sepsis | 3.87E-02 |
| **MYL9** | 10398 | 201058_s_at | 20q11.23 | Sepsis | 4.10E-02 |
| **GPR84** | 53831 | 223767_at | 12q13.13 | Sepsis | 4.34E-02 |
| **FGF13** | 2258 | 205110_s_at | Xq26.3 | Sepsis | 5.02E-02 |
| **RETN** | 56729 | 220570_at | 19p13.2 | Sepsis | 8.19E-02 |
| **CD177** | 57126 | 219669_at | 19q13.2 | Sepsis | 1.21E-01 |
| **OLFM4** | 10562 | 212768_s_at | 13q14.3 | Sepsis | 1.26E-01 |
| **MMP8** | 4317 | 207329_at, 231688_at | 11q22.3 | Sepsis | 1.65E-01 |
| **ALPL** | 249 | 1557924_s_at, 215783_s_at | 1p36.12 | Sepsis | 1.66E-01 |
| **CEACAM1** | 634 | 211889_x_at, 206576_s_at, 211883_x_at, 210610_at, 209498_at | 19q13.2 | Sepsis | 1.71E-01 |
| **BMX** | 660 | 206464_at | Xp22.2 | Sepsis | 1.75E-01 |
| **DEFA4** | 1669 | 207269_at | 8p23 | Sepsis | 1.80E-01 |
| **PRTN3** | 5657 | 207341_at | 19p13.3 | Sepsis | 3.74E-01 |
| **ELANE** | 1991 | 206871_at | 19p13.3 | Sepsis | 4.11E-01 |
| **FUT7** | 2529 | 210506_at, 217696_at | 9q34.3 | Sepsis (210506_at)  SIRS (217696_at) | 1.93E-01 |
| **SLC16A3** | 9123 | 202855_s_at, 202856_s_at, 217685_at, 217691_x_at, 213522_s_at | 17q25 | Sepsis, except 217685_at showed upregulation in SIRS | 1.39E-01 |
| **CAMK1D** | 57118 | 235626_at, 220246_at | 10p13 | SIRS | 1.00E-10 |
| **GPR183** | 1880 | 205419_at | 13q32.3 | SIRS | 9.32E-03 |
| **FAIM3** | 9214 | 221601_s_at, 221602_s_at | 1q32.1 | SIRS | 3.29E-02 |
| **ISG20** | 3669 | 33304_at, 204698_at | 15q26 | SIRS | 3.62E-02 |
| **LBH** | 81606 | 221011_s_at | 2p23.1 | SIRS | 3.74E-02 |
| **KLRB1** | 3820 | 214470_at | 12p13 | SIRS | 3.95E-02 |
| **PRKDC** | 5591 | 208694_at, 210543_s_at, 215757_at | 8q11 | SIRS | 1.00E-01 |
| **P2RY10** | 27334 | 1553856_s_at, 214615_at | Xq21.1 | SIRS | 1.10E-01 |
| **GNLY** | 10578 | 37145_at, 205495_s_at | 2p11.2 | SIRS | 1.20E-01 |
| **CCR7** | 1236 | 206337_at | 17q12-q21.2 | SIRS | 1.22E-01 |
| **LGALS2** | 3957 | 208450_at | 22q13.1 | SIRS | 1.26E-01 |
| **IL2RB** | 3560 | 205291_at | 22q13.1 | SIRS | 1.39E-01 |
| **ATP2B1** | 490 | 209281_s_at, 212930_at, 215716_s_at | 12q21.3 | SIRS | 1.64E-01 |
| **ITK** | 3702 | 211339_s_at | 5q31-q32 | SIRS | 1.72E-01 |
| **HLA-DQB1** | 3119 | 211654_x_at, 212999_x_at, 209480_at, 210747_at, 209823_x_at | 6p21.3 | SIRS | 1.77E-01 |
| **TREM1** | 54210 | 219434_at | 6p21.1 | SIRS | 1.77E-01 |
| **NLRC3** | 197358 | 236295_s_at | 16p13.3 | SIRS | 1.79E-01 |
| **TRBC1** | 28639 | 213193_x_at, 210915_x_at | 7q34 | SIRS | 1.81E-01 |
| **TXK** | 7294 | 206828_at | 4p12 | SIRS | 1.85E-01 |
| **GIMAP4** | 55303 | 219243_at | 7q36.1 | SIRS | 1.88E-01 |
| **HLA-DRA** | 3122 | 210982_s_at, 208894_at | 6p21.3 | SIRS | 1.90E-01 |
| **KLRF1** | 51348 | 220646_s_at | 12p13.31 | SIRS | 2.60E-01 |
| **FGFBP2** | 83888 | 223836_at | 4p16 | SIRS | 3.26E-01 |
| **P2RY14** | 9934 | 206637_at | 3q24-q25.1 | SIRS | 3.41E-01 |
| **CD2** | 914 | 205831_at | 1p13.1 | SIRS | 3.48E-01 |
| **KLRK1** | 22914 | 205821_at, 1555691_a_at | 12p13.2-p12.3 | SIRS | 3.75E-01 |
| **ALAS1** | 211 | 205633_s_at | 3p21.1 | Sepsis | 1.89E-01 |
| **HMBS** | 3145 | 203040_s_at | 11q23.3 | SIRS | 7.68E-01 |
| **TBP** | 6908 | 203135_at | 6q27 | SIRS | 7.34E-01 |
| * p-value is based upon the product of ANN rank order and Student t-test, with multiple corrections using Benjamini & Hochberg FDR. | | | | | |

**Table S2**

|  |  | **Adult** | | | **Paediatric** | | | | | | |
| --- | --- | --- | --- | --- | --- | --- | --- | --- | --- | --- | --- |
|  |  | **Sepsis** | **SIRS** | **Healthy** | **Sepsis** | **SIRS** | **Healthy** | **Septic-shock** | **SIRS-resolved** | **G+ve bacterial** | **G-ve bacterial** |
| **No. samples** | | 55 | 25 | 20 | 52 | 27 | 18 | 106 | 24 | 37 | 26 |
| **PCOLCE2** | Mean | 7.146372 | 5.972098 | 2.931815 | 4.188902 | 4.453569 | 3.325733 | 4.754249 | 3.865724 | 4.420893 | 4.081171 |
|  | Std dev | 1.797503 | 1.886693 | 0.16001 | 1.145141 | 1.578795 | 0.196085 | 1.544993 | 0.739343 | 0.586843 | 0.104795 |
|  | Median | 6.794587 | 5.002854 | 2.951806 | 3.848862 | 3.764816 | 3.310452 | 4.142451 | 3.776186 | 4.237329 | 4.092909 |
|  | Fold change | 2.43 | 2.03 | - | 1.25 | 1.33 | - | 1.42 | 1.16 | 1.32 | 0.53 |
| **FGF13** | Mean | 7.357857 | 6.125207 | 3.4505 | 5.489975 | 6.142282 | 4.072769 | 5.924819 | 5.007306 | 4.821494 | 4.903239 |
|  | Std dev | 2.348058 | 2.142222 | 0.323091 | 1.642439 | 1.563274 | 0.406377 | 1.635124 | 0.981686 | 0.239548 | 0.265531 |
|  | Median | 6.891794 | 5.323192 | 3.403083 | 5.223971 | 6.079241 | 4.101823 | 5.670775 | 4.713465 | 4.834425 | 4.905946 |
|  | Fold change | 2.13 | 1.77 | - | 1.34 | 1.50 | - | 1.45 | 1.22 | 1.18 | 1.20 |
| **MYL9** | Mean | 8.292942 | 7.676103 | 5.600875 | 6.442657 | 5.851603 | 5.710295 | 6.271361 | 6.085147 | 8.75357 | 7.674772 |
|  | Std dev | 1.416807 | 1.092403 | 0.564924 | 1.273861 | 0.994327 | 0.677948 | 1.567927 | 1.449964 | 1.553029 | 1.46254 |
|  | Median | 8.408008 | 7.664322 | 5.572423 | 6.36076 | 5.769825 | 5.714238 | 5.824588 | 5.680789 | 8.655715 | 8.05381 |
|  | Fold change | 1.48 | 1.37 | - | 1.12 | 1.02 | - | 1.09 | 1.06 | 1.53 | 1.34 |
| **SLC16A3** | Mean | 7.534278 | 7.092487 | 6.584696 | 6.919046 | 6.733126 | 6.19295 | 6.968403 | 6.704026 | 6.873497 | 6.563586 |
|  | Std dev | 0.528712 | 0.677104 | 0.247143 | 0.541177 | 0.638057 | 0.418304 | 0.511889 | 0.558002 | 0.456741 | 0.325802 |
|  | Median | 7.562614 | 7.093844 | 6.649347 | 6.949161 | 6.89148 | 6.121315 | 7.067651 | 6.852211 | 6.879259 | 6.648742 |
|  | Fold change | 1.14 | 1.07 | - | 1.11 | 1.08 | - | 1.12 | 1.08 | 1.10 | 1.05 |
| **CD177** | Mean | 11.5756 | 10.14491 | 5.497438 | 10.25227 | 10.24459 | 6.140925 | 11.12979 | 9.435331 | 8.226454 | 7.552127 |
|  | Std dev | 2.12961 | 2.568776 | 0.400497 | 1.808048 | 2.019692 | 0.954551 | 1.720012 | 1.551096 | 1.149626 | 0.414581 |
|  | Median | 11.86301 | 9.468328 | 5.434878 | 10.39305 | 11.0601 | 5.935937 | 11.39235 | 9.616595 | 7.755313 | 7.542503 |
|  | Fold change | 2.10 | 1.84 | - | 1.66 | 1.66 | - | 1.81 | 1.53 | 1.33 | 1.22 |
| **GPR84** | Mean | 9.807804 | 8.700051 | 4.995894 | 7.672993 | 8.037726 | 5.154432 | 8.505526 | 6.717829 | - | - |
|  | Std dev | 1.665561 | 1.536526 | 0.341247 | 1.468572 | 1.441047 | 0.624663 | 1.442548 | 0.999851 | - | - |
|  | Median | 9.845639 | 8.326931 | 4.979426 | 7.440086 | 8.414946 | 5.008798 | 8.740196 | 6.576508 | - | - |
|  | Fold change | 1.96 | 1.74 | - | 1.48 | 1.55 | - | 1.65 | 1.30 |  |  |
| **TDRD9** | Mean | 8.641945 | 7.734581 | 4.522221 | 6.994851 | 7.245498 | 5.032733 | 7.683913 | 6.484968 | - | - |
|  | Std dev | 1.111363 | 1.237282 | 0.302526 | 1.372653 | 1.352308 | 0.590322 | 1.558788 | 0.907127 | - | - |
|  | Median | 8.421598 | 7.829602 | 4.572278 | 6.999066 | 7.403158 | 4.897251 | 7.791969 | 6.348567 | - | - |
|  | Fold change | 1.91 | 1.71 | - | 1.38 | 1.43 | - | 1.52 | 1.28 |  |  |
| **KLRK1** | Mean | 7.989286 | 8.507953 | 7.84042 | 7.404418 | 7.481895 | 8.973588 | 7.073063 | 7.712912 | - | - |
|  | Std dev | 1.256702 | 1.287382 | 0.482944 | 1.081387 | 0.94235 | 0.528063 | 0.988994 | 0.880774 | - | - |
|  | Median | 8.292272 | 8.551995 | 7.958494 | 7.372864 | 7.206597 | 8.954718 | 6.929334 | 7.635857 | - | - |
|  | Fold change | 1.01 | 1.08 | - | 0.82 | 0.83 | - | 0.78 | 0.85 |  |  |

**Table S3**

| **Marker** | **Adult** | | | **Paediatric** | | | | | | |
| --- | --- | --- | --- | --- | --- | --- | --- | --- | --- | --- |
|  | **Sepsis** | **SIRS** | **Healthy** | **Sepsis** | **SIRS** | **Healthy** | **Septic-shock** | **SIRS-resolved** | **G+ve bacterial** | **G-ve bacterial** |
| **CD177** | 0.1229 | 0.0833 | 0.1780 | 0.1728 | 0.1673 | 0.1392 | 0.1701 | 0.1803 | 0.1369 | 0.1550 |
| **FGF13** | 0.1531 | 0.1066 | 0.2111 | 0.2170 | 0.2244 | 0.1450 | 0.2038 | 0.2107 | 0.1757 | 0.14780 |
| **GPR84** | 0.1056 | 0.1332 | 0.1827 | 0.1791 | 0.1597 | 0.1610 | 0.1692 | 0.1702 | - | - |
| **KLRK1** | 0.1303 | 0.1287 | 0.0873 | 0.1555 | 0.1090 | 0.0897 | 0.1640 | 0.1271 | - | - |
| **MYL9** | 0.1668 | 0.1942 | 0.1884 | 0.1708 | 0.1666 | 0.1679 | 0.1575 | 0.1286 | 0.1479 | 0.0987 |
| **PCOLCE2** | 0.1324 | 0.1438 | 0.1847 | 0.1838 | 0.1779 | 0.1774 | 0.1747 | 0.1957 | 0.0841 | 0.2163 |
| **SLC16A3** | 0.1677 | 0.1823 | 0.1499 | 0.1598 | 0.1534 | 0.1062 | 0.1861 | 0.1458 | 0.1547 | 0.1865 |
| **TDRD9** | 0.0541 | 0.1336 | 0.2109 | 0.1566 | 0.1711 | 0.1918 | 0.1505 | 0.2075 | - | - |

**Table S4**

| **Disease group** | **Patient cohort** | **No. genes** | **Overlapped genes** | **Non-overlapped genes** |
| --- | --- | --- | --- | --- |
| Sepsis | adult | 80 | - | - |
|  | paediatric | 80 | 5 | 75 |
|  |  |  |  |  |
| SIRS | adult | 80 | - | - |
|  | paediatric | 80 | 6 | 74 |
|  |  |  |  |  |
| Healthy | adult | 80 | - | - |
|  | paediatric | 80 | 9 | 71 |
|  |  |  |  |  |
| Septic-shock | paediatric | 80 | - | - |
| Sepsis | paediatric | 80 | 20 | 60 |
|  |  |  |  |  |
| SIRS-resolved | paediatric | 80 | - | - |
| SIRS | paediatric | 80 | 4 | 76 |
|  |  |  |  |  |
| G+ve bacterial | paediatric | 50 | - | - |
| G-ve bacterial | paediatric | 50 | 1 | 49 |

**Table S5**

|  |  |  | **Adult** | | | |
| --- | --- | --- | --- | --- | --- | --- |
| **Theme** | **Pathway description** | **PANTHER ID** | **Sepsis** | **SIRS** | **Healthy**  **Control** | |
| **Carbohydrate metabolism** | Fructose galactose metabolism | P02744 |  |  | |  |
|  | Glycolysis | P00024 |  |  | | 1 |
|  | Pentose phosphate pathway | P02762 |  |  | | 1 |
|  | TCA cycle | P00051 |  |  | | 1 |
| **Cell growth & death** | Apoptosis signalling pathway | P00006 |  |  | |  |
|  | p53 pathway | P00059--P04397--P04398 |  | 1 | |  |
| **Cell signalling** | Cadherin signalling pathway | P00012 | 1 |  | |  |
|  | FAS signalling pathway | P00020 |  |  | |  |
| **G protein-coupled receptors** | Adrenergic receptor signalling pathway | P04377--P04378--P04379 |  | 2 | | 2 |
|  | Dopamine receptor mediated signalling pathway | P05912 |  | 1 | | 1 |
|  | Endothelin signalling pathway | P00019 |  | 1 | | 1 |
|  | Gonadotropin releasing hormone receptor pathway | P06664 | 4 | 2 | | 1 |
|  | Histamine receptor signalling pathway | P04385--P04386 |  | 1 | | 1 |
|  | Opioid signalling pathway | P05915--P05916--P05917 |  |  | |  |
| **Growth & development** | Angiogenesis | P00005 | 2 | 1 | |  |
|  | Axon guidance | P00008--P00009 |  |  | |  |
|  | EGF receptor signalling pathway | P00018 | 3 | 2 | |  |
|  | FGF signalling pathway | P00021 | 2 | 1 | | 1 |
|  | Heme biosynthesis | P02746 |  |  | |  |
|  | PDGF signalling pathway | P00047 |  |  | |  |
| **Immune system** | B cell activation | P00010 |  |  | |  |
|  | Hypoxia response | P00030 |  |  | |  |
|  | Inflammation | P00031 | 3 | 4 | |  |
|  | Interleukin signalling pathway | P00036 |  | 2 | |  |
|  | T cell activation | P00053 |  |  | |  |
|  | Toll receptor signalling pathway | P00054 |  |  | |  |
| **Neurodegenerative diseases** | Alzheimer disease | P00003--P00004 |  |  | | 1 |
|  | Huntington disease | P00029 | 1 |  | |  |
|  | Parkinson disease | P00049 |  |  | |  |
| **Nucleotide metabolism** | De novo purine biosynthesis | P02738 |  |  | |  |
|  | Purine metabolism | P02769 | 1 | 1 | |  |
|  | Pyrimidine Metabolism | P02771 | 1 |  | |  |
| **Signal transduction** | 5HT type receptors signalling pathway | P04373--P04374--P04376 |  | 1 | | 1 |
|  | Angiotensin II-stimulated signalling | P05911 |  | 1 | |  |
|  | Hedgehog signalling pathway | P00025 |  |  | | 1 |
|  | Heterotrimeric G-protein signalling pathways | P00026--P00027--P00028 | 2 | 5 | | 1 |
|  | Integrin signalling pathway | P00034 | 3 | 2 | | 1 |
|  | mGluR pathways | P00039--P00040--P00041 |  | 3 | | 3 |
|  | p38 MAPK pathway | P05918 | 1 | 1 | |  |
|  | PI3 kinase pathway | P00048 |  |  | |  |
|  | Ras Pathway | P04393 | 2 | 1 | |  |
|  | TGF-beta signalling pathway | P00052 | 2 | 1 | |  |
|  | VEGF signalling pathway | P00056 | 1 | 1 | |  |
|  | Wnt signalling pathway | P00057 | 2 | 1 | | 1 |
| **Stress-related** | Endogenous Cannabinoid signalling | P05730 |  |  | |  |
|  | Oxidative stress response | P00046 |  |  | |  |
|  | Plasminogen activating cascade | P00050 |  |  | | 1 |
| **Miscellaneous** | Androgen/estrogene/progesterone biosynthesis | P02727 |  |  | |  |
|  | Blood coagulation | P00011 | 2 | 1 | | 1 |
|  | Cholesterol biosynthesis | P00014 | 1 | 1 | |  |
|  | Cortocotropin releasing factor receptor signalling pathway | P04380 |  |  | |  |
|  | Cytoskeletal regulation by Rho GTPase | P00016 | 1 | 1 | |  |
|  | DNA replication | P00017 |  |  | |  |
|  | DPP-SCW signalling pathway | P06212 |  |  | | 1 |
|  | Enkephalin release | P05913 |  | 1 | | 1 |
|  | GABA-B receptor II signalling | P05731 |  | 1 | | 1 |
|  | Insulin/IGF pathway | P00032--P00033 |  |  | |  |
|  | mAChR pathways | P00042--P00043 |  | 1 | | 1 |
|  | Nicotine degradation | P05914 |  |  | |  |
|  | Nicotine pharmacodynamics pathway | P06587 |  | 1 | |  |
|  | Nicotinic acetylcholine receptor signalling pathway | P00044 |  |  | | 1 |
|  | Oxytocin receptor mediated signalling pathway | P04391 |  |  | |  |
|  | Transcription regulation by bZIP transcription factor | P00055 |  |  | | 1 |
|  | Thyrotropin-releasing hormone receptor signalling pathway | P04394 |  |  | |  |
|  | Vitamin D metabolism and pathway | P04396 |  |  | |  |

**Table S6**

| **Overlapped Genes** | **Entrez ID** | **Cytoband** | | **Adult**  **Sepsis** | **Adult**  **SIRS** | **Adult Healthy** | **Paediatric Sepsis** | **Paediatric SIRS** | **Paediatric Healthy** | **Paediatric**  **SIRS-Resolved** | **Paediatric**  **Septic Shock** | **Paediatric Gram +ve Infection** | **Paediatric Gram -ve Infection** |
| --- | --- | --- | --- | --- | --- | --- | --- | --- | --- | --- | --- | --- | --- |
| **ALPL** | 249 | | 1p36.12 |  | CD177 |  |  |  |  |  | CD177 |  |  |
| **AMPD3** | 272 | | 11p15 | SLC16A3 | SLC16A3 |  |  |  |  |  |  |  |  |
| **ANKRD22** | 118932 | | 10q23.31 |  |  | GPR84 |  |  |  |  |  |  |  |
| **ATP9A** | 10079 | | 20q13.2 |  | GPR84 |  |  |  |  |  |  |  |  |
| **BST1** | 683 | | 4p15 |  | FGF13 |  | TDRD9 -- FGF13 |  |  |  |  |  |  |
| **C19orf45** | 374877 | | 19p13.2 |  |  | KLRK1 |  |  | KLRK1 |  |  |  |  |
| **C19orf59** | 199675 | | 19p13.2 |  | FGF13 |  | GPR84 --CD177 |  |  |  |  |  |  |
| **C1orf68** | 100129271 | | 1q21.3 |  |  | KLRK1 |  |  | KLRK1 |  |  |  |  |
| **CA4** | 762 | | 17q23 |  |  | SLC16A3 |  |  |  |  |  |  |  |
| **CD2** | 914 | | 1p13.1 |  | KLRK1 |  |  |  |  |  |  |  |  |
| **CD82** | 3732 | | 11p11.2 |  | GPR84 |  | PCOLCE2 -- SLC16A3 | SLC16A3 |  |  |  |  |  |
| **CAPN1** |  | |  |  |  |  |  |  |  | GPR84 |  |  |  |
| **CDADC1** | 81602 | | 13q14.2 |  | FGF13--GPR84 |  |  |  |  |  |  |  |  |
| **CEACAM1** | 634 | | 19q13.2 |  | FGF13--GPR84 |  |  | GPR84 |  |  | GPR84 | CD177 |  |
| **CMTM5** | 116173 | | 14q11.2 |  | MYL9 |  | MYL9 | MYL9 | MYL9 | MYL9 | MYL9 |  |  |
| **DDAH2** | 23564 | | 6p21.3 | CD177 |  |  | TDRD9 – GPR84 –  CD177 |  |  |  | CD177 |  |  |
| **DENND3** |  | |  |  |  |  | SLC16A3 |  |  |  | SLC16A3 |  |  |
| **DPY19L3** | 147991 | | 19q13.11 | TDRD9 |  |  |  |  |  |  |  |  |  |
| **ECRP** | 643332 | | 14q11.2 |  |  | TDRD9 |  |  |  |  |  |  |  |
| **ENTPD7** | 57089 | | chr10 | GPR84 |  |  | GPR84 |  |  |  | GPR84 |  |  |
| **EOMES** | 8320 | | 3p24.1 | KLRK1 |  |  |  |  |  |  |  |  |  |
| **EXOSC4** | 54512 | | 8q24.3 | CD177--GPR84--TDRD9 | GPR84 |  | GPR84 |  |  |  |  |  |  |
| **FLOT1** | 10211 | | 6p21.3 |  |  |  |  |  |  | SLC16A3 |  |  |  |
| **FUT7** | 2529 | | 9q34.3 | SLC16A3 | SLC16A3 |  |  |  |  | CD177 |  | CD177 |  |
| **FOLR3** |  | |  |  |  |  |  |  |  | TDRD9 |  |  |  |
| **GML** | 2765 | | 8q24.3 |  |  | SLC16A3 |  |  |  |  |  |  |  |
| **GOLGA1** | 2800 | | 9q33.3 | TDRD9 |  |  |  |  |  |  |  |  |  |
| **IDI1** | 3422 | | 10p15.3 | GPR84 | FGF13 |  |  |  |  |  |  |  |  |
| **IDNK** | 414328 | | 9q21.32 | TDRD9 | SLC16A3 |  |  |  |  |  |  |  |  |
| **IER3** | 8870 | | 6p21.3 |  |  | SLC16A3 |  |  |  |  |  |  |  |
| **ITGA2B** | 3674 | | 17q21.32 |  | MYL9 |  | MYL9 | MYL9 |  |  | MYL9 |  |  |
| **ITGB3** | 3690 | | 17q21.32 |  |  |  | MYL9 | MYL9 |  |  | MYL9 |  |  |
| **KCNE1** | 3753 | | 21q22.12 | FGF13 | TDRD9 |  |  |  |  |  |  |  |  |
| **LAPTM4B** |  | |  |  |  |  |  |  |  | PCOLCE2 |  |  |  |
| **LCN2** |  | |  |  |  |  |  |  |  | PCOLCE2 | GPR84 |  |  |
| **LOC100506175** | 100506175 | | chr20 |  |  | KLRK1 |  |  | KLRK1 |  |  |  |  |
| **LOC100507244** | 100507244 | | chr9 |  |  | KLRK1 |  |  | KLRK1 |  |  |  |  |
| **LOC439949** | 439949 | | 10p14 |  | KLRK1 |  |  |  |  |  |  |  |  |
| **MAP2K6** | 5608 | | 17q24.3 | FGF13 | TDRD9 |  |  |  |  |  |  |  |  |
| **MBD6** |  | |  |  |  |  | SLC16A3 |  |  |  | SLC16A3 | FGF13 |  |
| **MSRA** | 4482 | | 8p23.1 | GPR84 |  |  |  |  |  |  |  |  |  |
| **NECAB1** | 64168 | | 8q21.3 | CD177--GPR84 |  |  |  |  |  |  |  |  |  |
| **NRGN** |  | |  |  |  |  | MYL9 |  | MYL9 |  | MYL9 |  |  |
| **OLAH** | 55301 | | 10p13 |  | PCOLCE2--TDRD9 |  |  |  |  |  |  |  |  |
| **OSTalpha** | 200931 | | 3q29 | GPR84 | GPR84 |  |  |  |  |  |  |  |  |
| **PFKFB2** | 5208 | | 1q31 | TDRD9 |  |  |  |  |  |  |  |  |  |
| **PHF7** | 51533 | | 3p21.1 |  |  | KLRK1 |  |  | KLRK1 |  |  |  |  |
| **PLD1** | 5337 | | 3q26 | PCOLCE2 |  |  |  | TDRD9 |  |  |  |  |  |
| **PRDM5** | 11107 | | 4q25-q26 | CD177 | TDRD9 |  |  |  |  |  |  |  |  |
| **PRKAR2B** | 5577 | | 7q22 |  |  | MYL9 |  |  |  |  |  | MYL9 |  |
| **RETN** |  | |  |  |  |  |  |  |  | PCOLCE2 | GPR84 |  |  |
| **RGL4** |  | |  |  |  |  |  |  |  |  | CD177 |  |  |
| **SELP** |  | |  |  |  |  | MYL9 |  |  | MYL9 | MYL9 |  | MYL9 |
| **SIGLEC6** | 946 | | 19q13.3 |  |  | FGF13 |  |  | CD177 |  |  |  |  |
| **SLC22A8** | 9376 | | 11q11 |  |  | KLRK1 |  |  | KLRK1 |  |  |  |  |
| **TESK2** | 10420 | | 1p32 |  | MYL9 |  |  |  |  |  |  | FGF13 |  |
| **TGFB1L1** | 7041 | | 16p11.2 | MYL9 | MYL9 |  | MYL9 |  | MYL9 |  |  |  |  |
| **TREML1** | 340205 | | 6p21.1 |  | MYL9 | MYL9 | MYL9 | MYL9 |  | MYL9 | MYL9 |  |  |
| **ZDHHC19** | 131540 | | 3q29 | CD177--TDRD9 |  | KLRK1 | GPR84 |  | KLRK1 |  | GPR84 |  |  |

**Table S7**

| **Hub**  **Gene** | **CD177** | **FGF13** | **GPR84** | **KLRK1** | **MYL9** | **PCOLCE2** | **SLC16A3** | **TDRD9** |
| --- | --- | --- | --- | --- | --- | --- | --- | --- |
| **Hub Associated Genes** | MMP8  RETN | AMPD3  ARG1  ATP9A  BPI  C19orf45  C1orf68  CCR4  CCR7  CCR8  CD101  CD109  CD163L1  CD1A  CD1B  CD1E  CD200R1  CD207  CD209  CD244  CD276  CD2AP  CD2BP2  CD300LG  CD34  CD36  CD37  CD3EAP  CD4  CD40  CD40LG  CD44  CD47  CD53  CD59  CD5L  CD6  CD70  CD79B  CD80  CD81-AS1  CD84  CD8B  CD9  CD99L2  FLOT1  FUT7  GML  IDI1  IDNK  ITGA2B  ITGB3  KLRAP1  KLRC4  KLRG2  LOC100506175  MSRA  MYL9  PFKFB2  PHF7  PLD1  SIGLEC6  SLC22A8 |  | BTLA  CCR6  CD2  CD3D  CD3E  CD52  CD6  CD69  CD74  CD84  CD8A  CD8B  CD96  GIMAP4  GNLY  GPR183  IL2RB  ITK  KLRB1  KLRC4-KLRK1///KLRK1  KLRD1  LBH  NLRC3  P2RY10  TRBC1///TRBC2///TRBV19///TRBV3-1///TRBV5-4///TRBV6-5///TRBV7-2  TRBC1///TRBV19  TXK | AMPD3  ARG1  BPI///LOC149684  C19orf45  C1orf68  CCRL2  CD101  CD109  CD151  CD163L1  CD207  CD276  CD2AP  CD300A  CD300C  CD300LB  CD300LG  CD33  CD36  CD44  CD47  CD53  CD5L  CD68  CD79B  CD81-AS1  CD82  CD84  CD8B  CD9  CD99L2  CD99P1  CDADC1  FUT7  ITGA2B  ITGB3  KLRG2  PFKFB2  PHF7  PLD1  SLC22A8  TGFB1I1 | DACH1  METTL7B  ZDHHC19 | CD58  FLOT1 | CEACAM1  DACH1  FLOT1 |
