# Supplementary material for: Development of a Bioinformatics Framework for Identification and Validation of Genomic Biomarkers and Key Immunopathology Processes and Controllers in Infectious and Non-infectious Severe Inflammatory Response Syndrome
Source: Front Immunol. 2020 Mar 31;11:380. doi: 10.3389/fimmu.2020.00380 (PMC7147506; doi:10.3389/fimmu.2020.00380)
Supplement: Supplementary Information S4 — Description and discussion of gene interaction patterns in pediatric gram-negative and gram-positive bacteraemia. [file Table_4.DOCX]

**Supplementary Information S4**

**Gene Interaction Patterns in Paediatric Gram-Negative and Gram-Positive Bacteraemia**

Interaction maps of the core hub entities in paediatric bacteraemia are given in Supplementary Information S5, Figure S5.1 gram-positive bacteraemia and Figure S5.2 gram-negative bacteraemia. There are specific entity interactions with some of the core hub genes, similar to those observed with the other disease datasets, with little overlap in the complement of entities. The hub genes in paediatric gram-positive and gram-negative infection do not interact with each other or exhibit common shared interacting entities, with the exception of the MYL9-centred hub. This shares ALOX12 between the paediatric healthy control, paediatric gram-positive and paediatric gram-negative datasets and SELP, between the paediatric healthy control and paediatric gram-negative datasets. There are no interconnections between the hubs. This would imply very different underlying immune and other biological processes at play in the two respective infectious disease conditions. Interestingly ALOX12 (arachidonate 12-lipoxygenase) regulates platelet function (60) and is found associated with MYL9 in paediatric sepsis and resolved SIRS, but not any other control or disease group. This may reflect subtle differences in the MYL9-associated regulatory components between adults and children. There is an observed increased stimulatory activity across all the hubs in the gram-negative dataset and the MYL9-centred hub in the gram-positive dataset, highlighting again the prominence of this gene hub in most of the disease conditions in the analysis. There are negative regulatory profiles for the CD177, FGF13 and SLC16A3 hubs and a neutral stimulatory profile for the PCOLCE2-centred hub in the gram-positive dataset. Due to the absence of 3 of the key hubs (TDRD9, GPR84 and KLRK1) in this study, there seems little commonality between these interaction profiles and the other disease groups, or each other, therefore these were not discussed further.
