# Supplementary figures and images for: Development of a Bioinformatics Framework for Identification and Validation of Genomic Biomarkers and Key Immunopathology Processes and Controllers in Infectious and Non-infectious Severe Inflammatory Response Syndrome
Source: Front Immunol. 2020 Mar 31;11:380. doi: 10.3389/fimmu.2020.00380 (PMC7147506; doi:10.3389/fimmu.2020.00380)

**Supplementary Information S5**

**Figure S5.1**

**
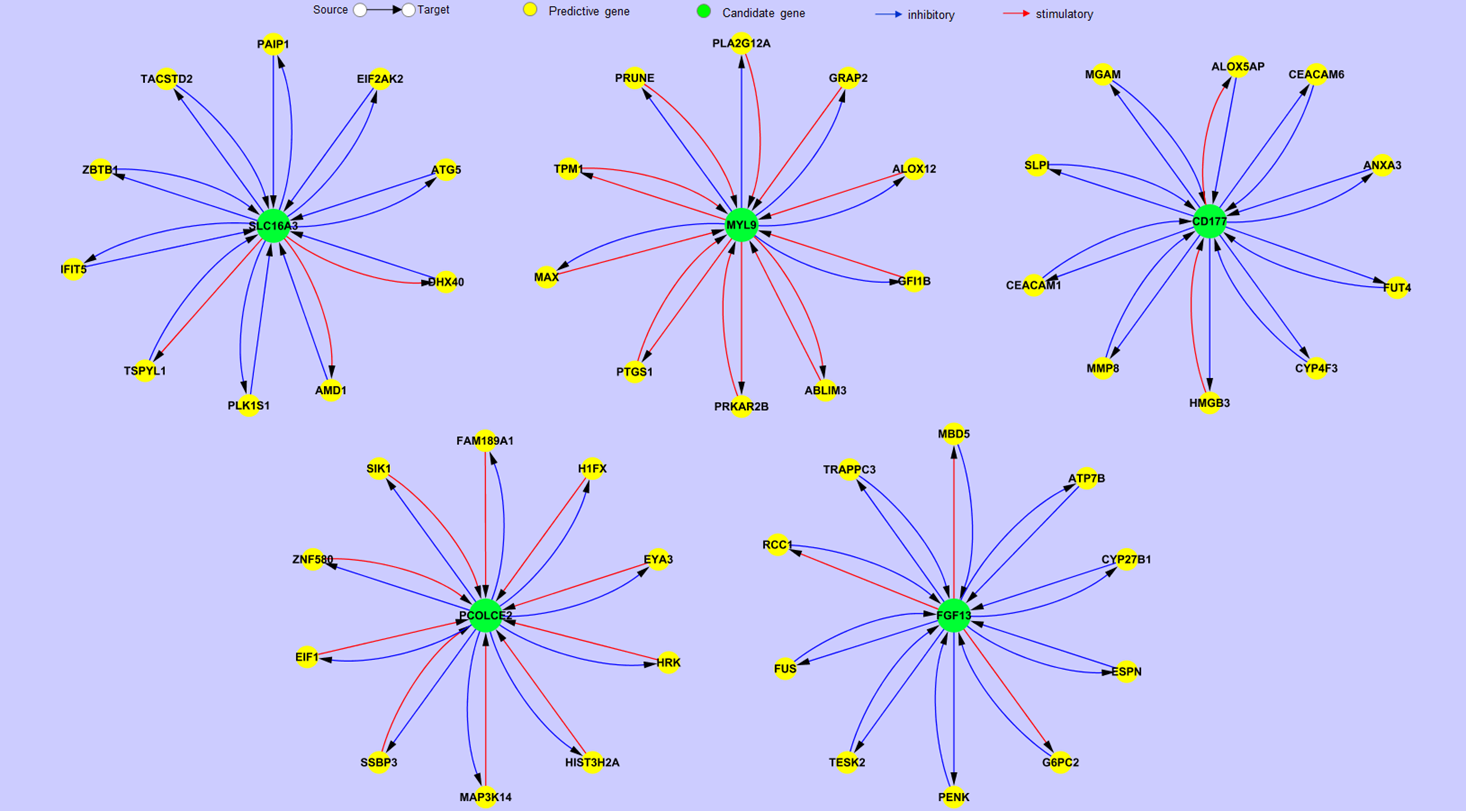
**

**Figure S5.2**

**
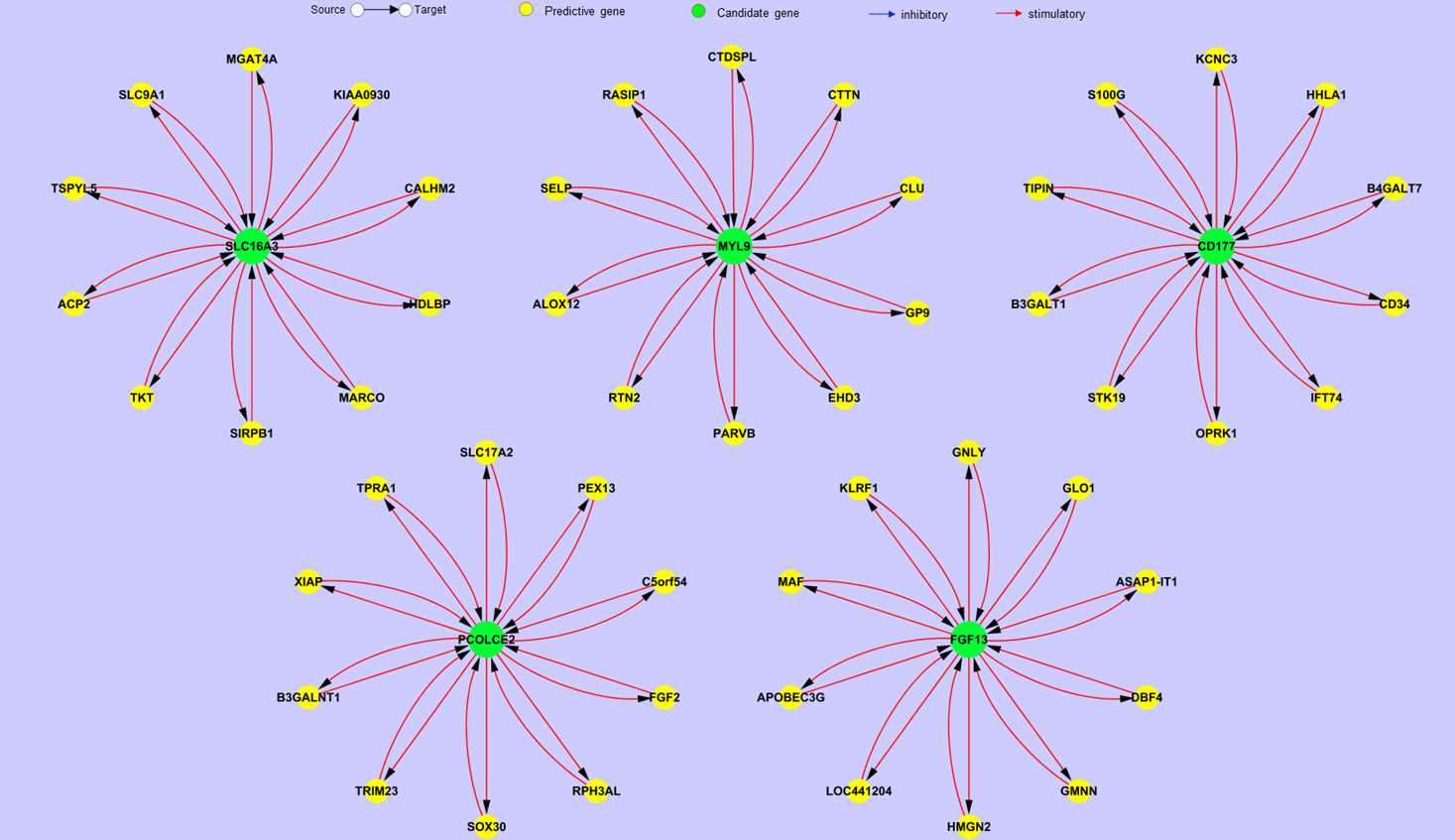
**

Supplement: Supplementary Information S5 — Figure S5.1: Hub Gene Entity Interaction Map for Pediatric Gram-Positive Bacteraemia. Figure S5.2. Hub Gene Entity Interaction Map for Pediatric Gram-Negative Bacteraemia. [file Table_5.DOCX]
